# Sprayable Polymer Blends With Short‐Chain Surface Segregation for Preventing Postoperative Abdominal Adhesions

**DOI:** 10.1002/adhm.202505289

**Published:** 2026-03-15

**Authors:** Robert J. Morris, Tejaswi Nori, Alex I. Halpern, Hannah LaPadula, Arthur V. Cresce, Sarah L. Wright, Anthony D. Sandler, Peter Kofinas

**Affiliations:** ^1^ Department of Chemical and Biomolecular Engineering University of Maryland College Park Maryland USA; ^2^ Sheikh Zayed Institute For Pediatric Surgical Innovation Joseph E. Robert Jr. Center for Surgical Care Children's National Medical Center Washington District of Columbia USA; ^3^ U.S. Army DEVCOM Army Research Laboratory Battery Science Branch Energy Sciences Division Adelphi Maryland USA

**Keywords:** adhesion barrier, sprayable polymer, surface segregation, tissue adhesive

## Abstract

Adhesions are common post‐surgical complications where fibrous tissue bridges adjacent surfaces following tissue injury. Although Seprafilm is a widely used clinical prophylactic, it often proves inadequate due to suboptimal mechanical stability and applicability. Solution Blow Spinning (SBS) enables the formation of polymer fibers tailored to complex geometries with excellent tissue adherence. Poly(D,L‐lactide‐*co*‐caprolactone) (PLCL) polymer has shown promise as an adhesions barrier but remains inconsistent because of its inherent tackiness and hydrophobicity. Here, high molecular weight (HMW) PLCL (40 or 80 kDa) was blended with low molecular weight (LMW) polyethylene glycol (PEG, 1 or 3 kDa). In vitro studies confirmed that PLCL/PEG fibers achieved tissue adhesion strengths above 10 kPa and sustained mechanical performance throughout clinically relevant degradation timelines. Blending PEG changes the surface composition of the spray‐deposited mats, substantially improving the hydrophilic character and lowering protein adherence in vitro. In a murine cecal ligation model, the PLCL/PEG blends demonstrated significantly reduced adhesion severity and incidence compared to both untreated and Seprafilm‐treated controls.

## Graphical abstract

Adhesions’ high occurrence rates and high morbidity render them a critical challenge to be addressed. Current prevention methods, such as physical barriers, have many limitations, resulting in inconsistent safety and efficacy. This study demonstrates the potential for sprayable polymeric materials as an adhesion barrier. These materials demonstrate high tissue‐adhesion, biocompatibility, and anti‐fouling capabilities, offering a unique approach of preventing adhesions postoperatively.

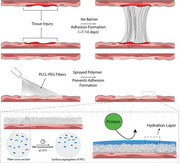

## 1 Introduction

Adhesions are medical complications that arise from uncontrolled fibrinogenesis following disruptions of the mesothelial linings, leading to unintended connection of tissue surfaces after surgeries, internal trauma, and ischemia [1, 2]. Adhesions are particularly prevalent after abdominal procedures with staggering occurrence rates exceeding 95%, and these fibrotic bridges can evolve into dense scar tissue resulting in numerous life‐threatening complications and substantial patient and healthcare costs [3, 4]. In the peritoneal cavity, adhesions are documented as the leading cause of secondary female infertility and small bowel obstruction, where severe cases can result in mortality rates of up to 10% [5, 6, 7, 8]. It is estimated that over 20 million patients in the United States develop adhesions every year [9]. Despite not every case resulting in immediately life‐threatening health concerns, adhesion formation can result in severe detriment of patient quality of life via chronic pain or paralysis; the development of which can result in psychological disorders and suicide [10]. In conjunction with the extreme prevalence rates, this results in a necessity for clinical intervention to treat adhesions, creating extensive economic, labor, and patient burdens across the globe.

The pathology of adhesions involves a complex interplay of biological mechanisms but is generally associated with an imbalance between fibrin deposition and degradation during the wound healing process [5]. Under normal conditions, injured tissue surfaces heal via secretion of numerous compounds, including coagulants, pro‐inflammatory cytokines, and growth factors, which contribute to the formation of a fibrous matrix over several days. This matrix is subsequently degraded via fibrinolysis. In adhesion formation, this fibrinolytic activity is suppressed and overtaken by inflammation and coagulation processes. These processes enhance fibrin deposition through the expression of cytokines and macrophages and the conversion of fibrinogen to fibrin by thrombin [11]. Over the following weeks, the development of vasculature and scar tissue within the adhesions significantly strengthens them, escalating the risks to patients [12, 13]. This mechanistic complexity results in therapeutic treatment approaches being impractical, further compounded by risks of impacting the progression of wound‐healing post‐operatively.

Surgical intervention to remove and sever adhesions, known as adhesiolysis, can be performed open or via laparoscopy. These procedures are typically reserved for patients with high‐risk obstructive complications and are rarely employed for non‐life‐threatening conditions [14]. Adhesiolysis has a limited long‐term efficacy, with rates of recurrent adhesive small bowel obstruction after adhesiolysis of 18% after 10 years and 29% after 30 years [15]. Additionally, adhesiolysis imposes a significant burden on both patients and the health care system, with inpatient expenditures totaling $2.3 billion following adhesiolysis‐related procedures in 2005 [14].

The economic burden of adhesions is substantial, with annual healthcare costs exceeding $3 billion for hospitals and over $2 billion for patients treated in the United States alone [4]. Given the high expense and limited efficacy of adhesiolysis, there is a critical need for effective preventive measures. Currently, the primary products utilized for preventing adhesions include Seprafilm (Genzyme), a dried carboxymethylcellulose sheet, and Interceed (Johnson & Johnson), a woven oxidized regenerated cellulose mat. However, these materials have significant limitations, including rapid degradation, brittleness, difficulty with application, and poor adhesion to wet tissues [15, 16, 17, 18, 19, 20, 21, 22, 23]. These shortcomings result in inconsistent and suboptimal performance in preventing adhesions, emphasizing the necessity for more reliable and effective solutions.

Current research into novel adhesion barriers focuses on films, hydrogels, and fibers that are tailored to prevent adhesions either through material design or by inclusion of a therapeutic molecule. A significant portion of this research focuses on developing drug‐delivery materials to deliver a variety of therapeutic molecules that prevent adhesions by regulating parts of the complex adhesion formation mechanism [24, 25, 26]. On the other hand, materials can also be designed to act as physical barriers to isolate the wound surface, a strategy employed in both research and commercially developed adhesion barriers. These materials are often designed to possess anti‐fouling properties, achieved by improving the material's hydrophilicity or creating superhydrophobic surfaces [27, 28, 29]. Often, this would imply utilizing complex chemistries to attach hydrophilic groups to the surface or surface patterning to induce superhydrophobicity, both of which hinder translatability and scalability.

One methodology to overcome the shortcomings of current adhesion barriers is by the deposition of polymeric fiber mats onto tissues through solution blow‐spinning (SBS). This methodology is optimal for material deposition within the body, with individual fibers able to substantially form and mimic the curved organ and tissue surfaces of human anatomy. With an application performed by an airbrush that can be adjusted for pressure and other flow parameters, such as nozzle diameter, these sprayed polymer fibers offer flexibility and ease of use for surgeons who can rapidly deploy a material onto any tissue surface. Furthermore, by blending polymers with different molecular weights, SBS can generate fiber mats that have degradation tuned to align with abdominal wound healing.

Our research group has previously employed SBS for various surgical applications, including burn wound dressings, abdominal sealants, and hemostatic materials [30, 31, 32, 33, 34]. In initial adhesions‐related investigations, we used SBS to deposit a poly(lactic‐co‐glycolic) acid (PLGA) barrier that ultimately proved inflammatory due to acidic byproducts of PLGA degradation. With inflammation resulting in increased adhesions formation, we then used SBS to spray a blend of high and low molecular‐weight (HMW/LMW, 40 kDa/5 kDa) poly(D,L‐lactide‐*co*‐caprolactone) (PLCL). This composition allowed for controlled surface erosion and reliable wet tissue adherence, enabling the material to gradually erode and potentially dissolve incipient tissue connections before they mature into full adhesions [35]. Although promising, the standalone efficacy of this material was limited, necessitating the incorporation of a small‐molecule therapeutic to significantly reduce adhesion formation [36]. In general, however, relying on a pharmacological agent raises concerns about systemic toxicity and the potential to disrupt normal wound‐healing processes, especially given the complex biology driving adhesion formation [37, 38, 39]. Consequently, there is a pressing need for a drug‐free, biocompatible, and biodegradable sprayable material that can prevent adhesion formation without risking patient safety or impeding healthy tissue repair.

One way to prevent adhesion formation would be to prevent the deposition or migration of fibrinogenic and angiogenic molecules present in the initial stages with the help of a physical barrier. As a result, this material must possess properties that allow it to resist the deposition of proteins on the surface. Historically, polyethylene glycol (PEG), a polymer found in numerous FDA‐approved medical devices, has been used as an anti‐fouling material that can resist protein adsorption, mitigating the foreign body response of implanted biomaterials [40, 41, 42, 43]. This anti‐fouling ability of PEG is a result of its hydrophilicity, which creates a “hydration layer” on the surface of the materials, preventing protein adsorption, extending in vivo retention times.

In this work, we studied the impact of blending HMW PLCL (40 or 80 kDa) with LMW PEG (1 or 3 kDa) to enhance the efficacy of a sprayable adhesion barrier. Although PLCL blends offer tunable degradation rates conducive to clinically relevant biodegradability, their limited hydrophilicity and surface tackiness previously necessitated the addition of pharmacological agents to achieve optimized adhesion prevention. To overcome these challenges, we explored the blending of LMW PEG with HMW PLCL to induce PEG surface segregation, favoring the LMW hydrophilic PEG on the material surface. The shorter LMW PEG chains preferentially migrate to the surface, driven by entropic and interfacial energy factors. This surface enrichment of LMW species reduces the overall free energy of the system. By coupling PLCL's controlled erosion profile with PEG's inherent hydrophilicity, we aimed to create a biocompatible barrier capable of maintaining robust tissue separation and minimizing fibrinogenic molecule adsorption.

Our approach began with in vitro evaluations of mechanical properties and degradation profiles to identify optimal PLCL/PEG blend ratios. We then performed x‐ray photoelectron spectroscopy to determine surface chemistry and protein adsorption assays to confirm the surface properties imparted by PEG. Finally, we tested selected blends in a murine cecal ligation model of abdominal adhesions and conducted immunological analyses to validate their performance and safety in vivo. These combined efforts aimed to establish a spray‐deposited, wet‐tissue‐conforming, hydrophilic adhesion barrier that obviates the need for therapeutic agents while still providing effective adhesion prevention.

## 2 Materials and Methods

### 2.1 Polymer Solution Preparation

Polymer blends were prepared in ethyl acetate at a consistent 20% (w/v) concentration. Ethyl acetate, a Class III solvent with low vapor pressure, is approved by the FDA and the International Council for Harmonisation as having minimal toxicity. Two distinct molecular weights of poly(D,L‐lactide‐*co*‐caprolactone) (PLCL) served as the high molecular weight (HMW) component: 80 kDa (70:30 LA:CL, acid endcap, Mn 75,000–85,000; Akina) and 40 kDa (70:30 LA:CL, acid endcap, Mn 35,000–45,000; Akina). The low molecular weight (LMW) fraction consisted of polyethylene glycol (PEG) at either 950–1050 Da or 3,350 Da (Sigma–Aldrich). HMW:LMW mass ratios were formulated at 70:30 or 90:10. PEG content was capped at 30% by polymer mass, as higher PEG levels prevented uniform fiber mat formation during spraying. Spray deposition was performed using an airbrush (Master Airbrush, G222‐SET, 0.2 mm nozzle) connected to a pressurized CO_2_ tank regulated at 20 psig. This setup facilitated the application of conformal fiber mats onto target substrates.

### 2.2 Pull‐apart Adhesion Testing

Pull‐apart adhesion measurements were conducted on a TA Instruments DMA Q850. Ten‐millimeter square sections of frozen porcine intestine were thawed and equilibrated to room temperature by lightly wetting the tissue and allowing it to warm for 10 min in 37°C ambient air. Each tissue piece then received 1 mL of the polymer solution, which was left to set for 15 min at 37°C. Two polymer‐coated samples were brought into contact and affixed to the instrument clamps using superglue. The DMA operated in compression mode, applying a 1 N preload for five minutes. After this, a controlled force ramp at 0.5 N/min was applied until the samples separated. The peak force at failure was recorded as the adhesion strength, along with the specific mode of failure. Each sample set was tested in quintuplicate (n = 5).

### 2.3 Tensile Testing

Tensile tests were performed to track changes in mechanical properties as the sprayed polymer samples degraded. Undegraded samples (0‐day timepoint) were generated by spraying 2 mL of polymer solution onto a petri dish. For subsequent timepoints (1, 3, 5, 7, 10, and 14 days), samples were removed from their degradation medium (described in ***Mass loss and degradation testing***), cut to 10 mm × 5 mm, and measured precisely before testing. A TA Instruments DMA Q850 equipped with a film tension clamp was used under tension mode, applying a controlled force ramp from 0 to 1 N at 0.05 N/min with a 0.2% offset to account for toeing. Each sample was tested in quintuplicate (n = 5).

### 2.4 Scanning Electron Microscopy

Polymer samples were prepared by spraying approximately 0.1 mL of solution (20% w/v) directly onto double‐sided carbon conductive tape. This method reduced the handling of the polymers to not to impact morphology before imaging. SEM imaging was performed on a Thermo‐Fisher Scientific Phenom Pro operating in low vacuum (∼ 60 Pa pressure) mode with an operating distance of 5 mm. Beam voltage was set at 15 kV for imaging, and an adaptive imaging algorithm was used to stabilize the image area during the acquisition period. Scale bars were determined automatically by software analysis.

### 2.5 Mass Loss and Degradation Testing

Polymer samples were produced by spraying 2 mL of solution (20% w/v) onto a 60 mm glass petri dish using solution blow spinning (SBS). The airbrush (Master Airbrush, G222‐SET, 0.2 mm nozzle diameter) was held approximately 10 cm above the dish, and a Sartorius ME‐5 microbalance was used to measure the net increase in mass post‐spraying (initial mass, *m_i_*). Each sample was submerged in 10 mL of 1× PBS and incubated at 37°C. Samples were collected at 1, 3, 5, 7, 10, and 14 days, then dried in a vacuum desiccator for three days. The final mass, *m_f_*, was recorded to calculate mass loss percentage defined as $\frac{m_{i}-m_{f}}{m_{i}}\cdot 100$. Each timepoint and composition was tested in quintuplicate (n = 5).

### 2.6 X‐ray Photoelectron Spectroscopy

X‐ray photoelectron spectroscopy (XPS) measurements were performed using a PHI 5000 VersaProbe III Photoelectron Spectrometer with monochromated Al K*α* X‐rays at 1486.6 eV. Polymer samples were prepped as described in ***Mass loss and degradation testing***, removed from the glass dishes, and dried in a glovebox antechamber at 40°C for 24 h to remove excess water. Polymer samples were then transferred to the XPS in a plastic jar filled with desiccant so that the samples were not exposed to ambient atmosphere at any time. XPS binding energy data was analyzed with PHI MultiPak software using a smart (non‐Shirley) baseline. XPS analysis was performed by removing the background signal and calibrating the C‐C C1s main peak to 284.8 eV. The composite peaks were decomposed using fixed FWHM parameters after the background subtraction and shifted main peak.

### 2.7 Water Contact Angle Measurements

Surface hydrophilicity of undegraded (day 0) and degraded samples was evaluated using a sessile drop method. A 10 µL droplet of deionized (DI) water was placed on the sample surface, and images were captured with a Canon EOS1200D camera. Contact angles were quantified in ImageJ (National Institutes of Health). The polymer samples were prepared as described in ***Mass loss and degradation testing***, and five replicates (n = 5) were measured for each composition and timepoint.

### 2.8 Protein Adsorption

Protein adsorption was evaluated by incubating polymer‐coated coverslips with a 10 mg/mL bovine serum albumin (BSA) solution. Coverslips (22 mm diameter) were sprayed using 2 mL of polymer solution, placed into a 6‐well plate, and submerged in 4 mL of BSA solution. Samples were then incubated at 37°C with shaking (150 RPM) for one hour. Post‐incubation, the supernatant's absorbance was measured at 278 nm on a QuickDrop Spectrophotometer (SpectraMax) and compared to a BSA standard curve to determine the remaining protein in solution (Figure S1). Adsorbed protein mass was calculated by subtracting the remaining amount of BSA from the original. Background readings, including BSA adsorption to well surfaces without polymer, were subtracted out. Five samples (n = 5) were used per polymer composition.

### 2.9 Mouse Cecal Ligation Adhesion Model

All animal protocols were approved by the Children's National Hospital Institutional Animal Care and Use Committee (IACUC #000030703) and followed PHS guidelines, the NIH Guide for the Care and Use of Laboratory Animals, and the Animal Welfare Act.

#### 2.9.1 Animals

Twenty C57BL/6 female mice (6–10 weeks old, Jackson Laboratory) were randomly allocated to four groups (n = 5/group): untreated (negative control), Seprafilm (clinical control), or SBS‐coated cecum with either an 80 kDa PLCL/3 kDa PEG blend or a 40 kDa PLCL/1 kDa PEG blend.

#### 2.9.2 Procedure

Anesthesia was induced with 5% isoflurane in an induction chamber and maintained at 2–3% via nose cone. A 1 cm midline laparotomy was performed to expose the cecum, which was ligated approximately 0.5 cm from its distal tip with 5–0 Vicryl suture. For negative controls, the cecum was returned to the abdominal cavity after two minutes. For the Seprafilm control, a 1 cm^2^ piece of Seprafilm was placed on the ligated cecum. The polymer treatment groups received 2 mL of polymer solution sprayed directly onto the ligated cecum prior to closure. The peritoneum and skin were closed with 5–0 Vicryl, and 0.1 mg/kg buprenorphine was administered postoperatively.

#### 2.9.3 Endpoint

Eight days postoperatively, animals were euthanized. A blinded surgeon scored adhesion severity and frequency based on a modified Mazuji scale and noted any signs of inflammation or polymer residue.

### 2.10 Histological Analysis

On postoperative day eight, ligated cecal tissues were formalin‐fixed (10% neutral buffered formalin), paraffin‐embedded, sectioned at 5 µm, and stained with hematoxylin and eosin (H&E) (Histoserv Inc.). Digital images were acquired at 10x and 40x magnifications (cellSens, Olympus). For cellularity assessments, five high‐resolution 40x fields per section were analyzed in ImageJ. After calibration to 1 µm/pixel and conversion to an RGB stack, a threshold of 100 was set, and the purple‐stained region (% area) was measured. These values were averaged for each mouse.

### 2.11 Wound Healing Gene Expression

RNA was isolated from frozen cecal tissue using Trizol reagent (Life Technologies, Frederick, MD), and 6 µg RNA was reverse‐transcribed with the High‐Capacity cDNA Reverse Transcription Kit (Life Technologies). Quantitative PCR was performed using TaqMan Gene Expression Master Mix (Life Technologies) on a QuantStudio7 Flex RT‐PCR system (Thermo Fisher Scientific, Waltham, MA), according to the manufacturer's instructions. Each target gene was quantified in triplicate, including no‐template and endogenous control using GAPDH. Gene‐specific assays were Mm00434228_m1 for Il1b, Mm0046190_m1 for IL‐6, Mm00443258_m1 for TNF‐α, Mm00437306_m1 for VEGF‐A, Mm01178820_m1 for TGFβ‐1, Mm00433287_m1 for FGF‐2, Mm00801666_g1 for COL1a1, Mm00802305_g1 for COL3a1, and Mm99999915_g1 for GAPDH (Life Technologies, Thermo Fisher). Changes in relative gene expression normalized to GAPDH levels were determined using the ΔΔCt method. First, the difference between the Ct values (ΔCt) of the gene of interest and the housekeeping gene was calculated for each sample. Then the ΔCt values for the control samples were averaged. The difference in the ΔCt values between each experimental sample and the control sample (ΔΔCt) was calculated. The fold‐change in expression of the gene of interest compared to the housekeeping gene for each sample was calculated as 2−ΔΔCt, and the results were averaged for graphical representation.

### 2.12 Statistical Analysis

Data were analyzed using Origin (OriginLab). One‐way ANOVA followed by Tukey's post hoc test was performed to identify statistically significant differences among groups. Error bars represent the standard error (SE). Asterisks indicate significance (*p < 0.05; **p < 0.01; ***p < 0.001). Letter labeling was occasionally used; groups sharing the same letter are not statistically different, while different letters denote significant differences within a defined confidence interval. No datasets or animals were excluded from analysis, and sample sizes were chosen to achieve at least 95% power in detecting significant effects.

## 3 Results

### 3.1 Mechanical Properties, Biodegradation, and Surface Characteristics of PLCL/PEG Blends

**FIGURE 1:**
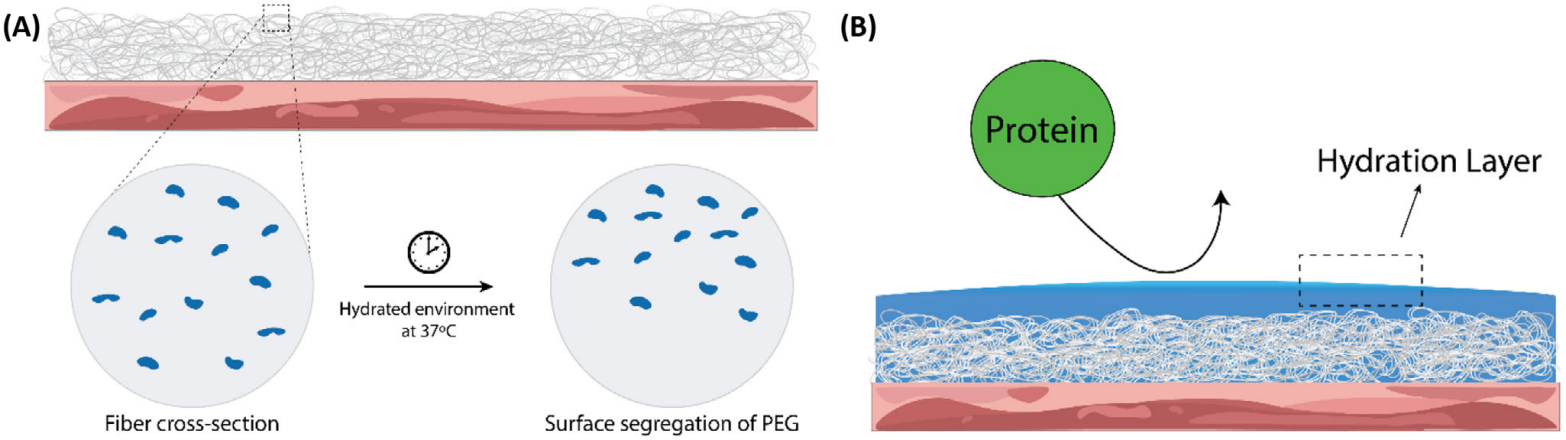
Schematic of polymer phase separation and protective mechanism. (A) Upon spraying, low molecular weight, hydrophilic PEG segregates from the higher molecular weight, hydrophobic PLCL toward the surface. (B) Surface‐localized PEG absorbs water and forms an aqueous layer, preventing adsorption of proteins and other biomacromolecules.

Blending polymers of distinct molecular weights offers a strategy to create sprayable materials with adjustable degradation profiles. In these systems, the LMW component degrades relatively quickly, segregating to the outer layer of the polymer fiber mat (Figure 1A), while the HMW fraction exhibits slower, surface‐eroding behavior. When LMW PEG is incorporated into PLCL fibers, its presence is expected to enhance the barrier's anti‐adsorption function via a fostered hydration layer on the polymer fiber mat surface, wherein bound water creates a steric barrier that deters hydrophilic proteins from binding (Figure 1B). In this system of PLCL/PEG, the phase separation by surface segregation is not just due to molecular weight differences, but the immiscibility of the polymers in this binary system. By leveraging PEG as the LMW fraction, the material helps maintain a consistently hydrophilic surface over the clinically relevant degradation window, further preventing unwanted protein interactions. Meanwhile, HMW PLCL provides robust mechanical integrity, conformal spraying capability, and inherent biocompatibility.

Adhesions generally form over a 7‐day period postoperatively that can extend up to two weeks. Potential physical barrier materials must continue to prevent contact between tissue surfaces over this timespan, which is critical for sufficient wound healing, particularly of the mesothelial layer. To account for this, degradation of the surface eroding PLCL/PEG blends was analyzed at 37°C while submerged within phosphate‐buffered saline (PBS) over a two‐week span. This was supplemented by measuring the tensile stiffness of these degraded samples to determine the material robustness over this time span.

**FIGURE 2:**
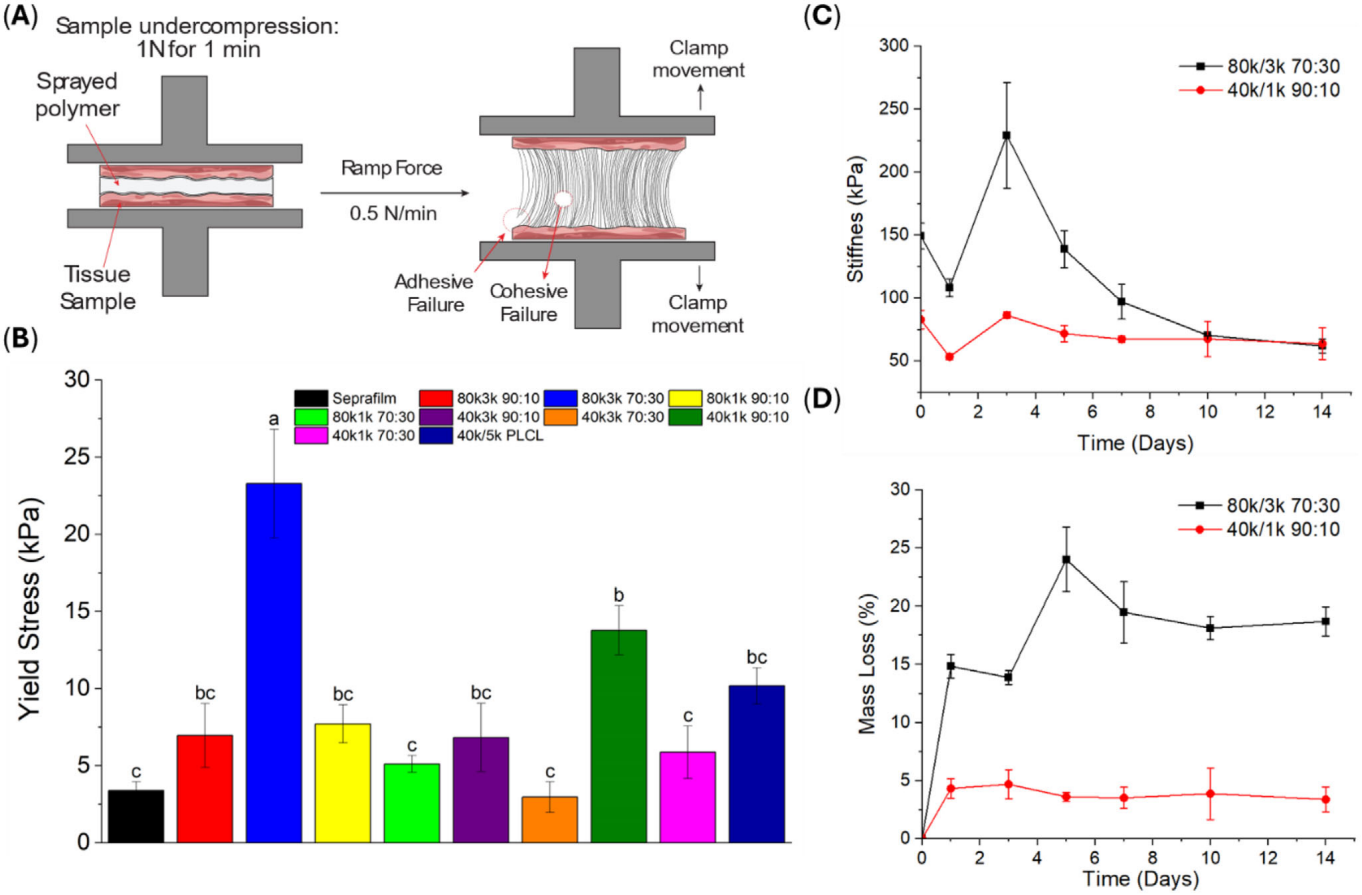
Mechanical characterization and degradation profiles of sprayed polymer blends. (A) Schematic of pull‐apart adhesion testing showing both adhesive and cohesive failure modes. (B) Initial (day zero) wet‐tissue pull‐apart adhesion scores for sprayed polymer blends. (C) Tensile stiffness of the two selected polymer blends during in vitro degradation. (D) Mass loss profiles of sprayed polymer blends over 14 days of in vitro degradation. All data are represented as mean ± SE. Different letters between groups indicate statistically significant differences at a 0.05 significance level.

Robust wet‐tissue adhesion, along with reliable mechanical strength and controlled degradation over time, are essential characteristics for an adhesion barrier. We initially assessed each blend via pull‐apart adhesion testing, where each was sprayed onto wet porcine intestinal tissue, compressed, and then pulled apart with a dynamic mechanical analyzer (DMA) in tension mode to measure yield stress and document failure mode (Figure 2A and Figures S2,S3). When compared to Seprafilm, a standard clinical control, all blends tested exhibited elevated ex vivo tissue binding strength, regardless of the molecular weight combination or polymer ratio (Figure 2B). Notably, each blend showed high wet‐tissue adhesion values (>1 N/cm^2^ or >10 kPa), in contrast to neat 40 kDa and 80 kDa PLCL, which measured 3 and 9 kPa, respectively [35]. Although no clear pattern emerged across all tested compositions, the 80k/3k (70:30) and 40k/1k (90:10) PLCL/PEG blends stood out for their notably higher tissue adhesion strengths, especially when compared to our previously published blend of 40 and 5 kDa PLCL. In particular, the high proportion of PEG in the 80k/3k (70:30) blend promotes water absorption from wet tissue surfaces, resulting in enhanced interfacial hydrogen bonding and superior adhesion [44, 45]. In the 40k/1k (90:10) blend, the 1 kDa PEG fraction melts at body temperature (37°C), prompting a fiber‐to‐film transition that enlarges the interface between the material and underlying tissue (Figures S4–S6). This effect leads to the remarkable adhesion strength observed. Due to their enhanced tissue‐binding properties, the 80k/3k (70:30) and 40k/1k (90:10) blends were selected for further characterization.

Over time, in vivo swelling and degradation alter the mechanical behavior of these polymer blends. To examine these changes, tensile properties were measured using a dynamic mechanical analyzer (DMA) in tensile mode. The 80k/3k (70:30) and 40k/1k (90:10) blends initially possessed yield stresses of ∼150 and ∼80 kPa, respectively, which declined steadily across 14 days of degradation (Figure 2C). Despite the drop, the 40k/1k (90:10) blend remained mechanically robust during this period. Mass loss data further illustrates these differences: over 14 days, the 80k/3k (70:30) blend lost more than 20% of its mass, whereas the 40k/1k (90:10) blend lost only about 5% (Figure 2D). This relatively slower degradation rate in the 40k/1k blend correlates with its consistent stiffness over the two‐week interval, while the 80k/3k blend shows a substantial drop in stiffness over the same timeframe.

**FIGURE 3:**
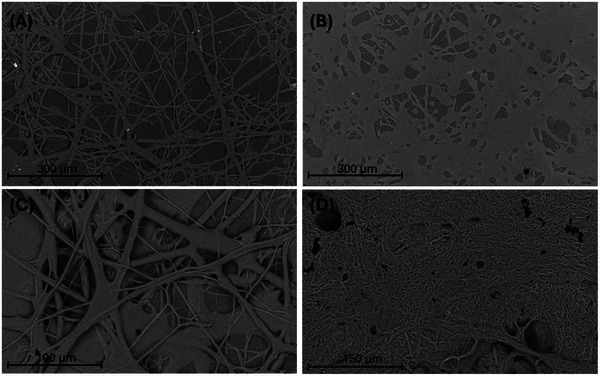
Visual analysis of neat PLCL and blended PLCL/PEG. Scanning electron microscopy was used to image (A) 40 kDa PLCL, (B) 40k/1k (90:10), (C) 80 kDa PLCL, and (D) 80k/3k (70:30). Blending PEG into neat PLCL results in a transition from dense fibers to more uniform films after spraying upon heating.

To visualize this transition, scanning electron microscopy of the neat PLCL and blended PLCL/PEG fibers was performed. Spraying neat 40 kDa PLCL results in thin overlapping lattices of fibers that form junctions for cohesive stability (Figure 3A). By blending in 1 kDa PEG, forming the 40k/1k (90:10) blend, it is apparent that the blend melts into a uniform thin film that loses the layering, demonstrating the resulting surface conformation upon the porcine tissues (Figure 3B). The neat 80 kDa PLCL fibers are noticeably more robust, with denser layering and interweaving between them (Figure 3C). Upon addition of the 3 kDa PEG, forming the 80k/3k (70:30) blend, the fiber morphology significantly changes, possibly due to increased crystallinity in the fiber matrix due to addition of 3 kDa PEG (Figure 3D). There are still some fibers present, unlike the 40k/1k (90:10) blend and this is likely due to the higher melting point of the 3 kDa PEG, resulting in an incomplete uniformity of the transitioned fibers. These trends are exhibited in the other tested polymer blends, with the 70:30 ratios exhibiting greater porosity due to the lower mass of PLCL in the spray (Figure S7A–H). Although the observed trends of the changing fiber morphology with PEG inclusion can be attributed to varying degrees of PLCL/PEG phase separation, it is important to note that the overall morphology is likely heterogeneous considering the 3D structure of the sprayed mats.

**FIGURE 4:**
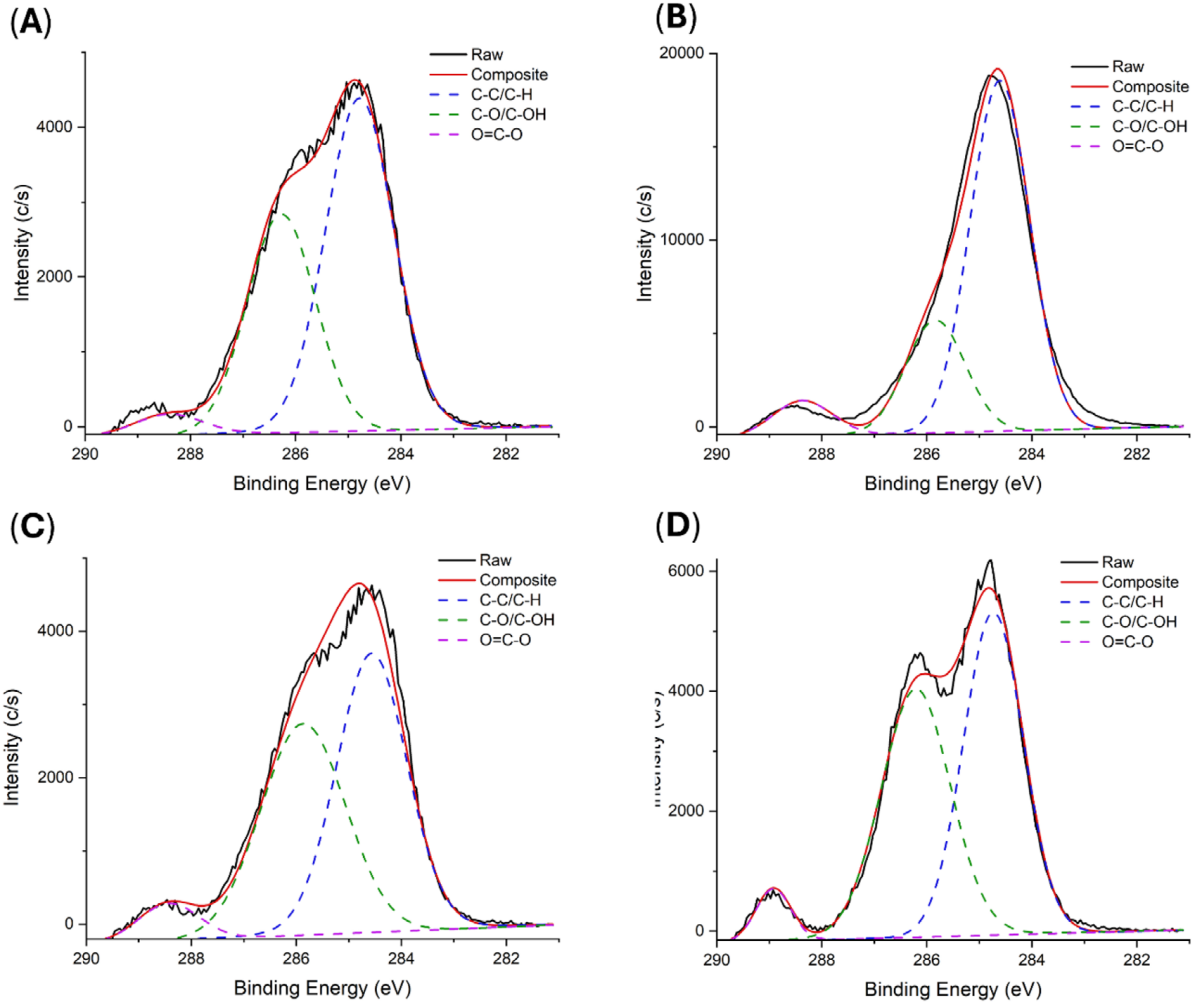
XPS analysis of sprayed polymer blend surface chemistry. (A) Neat PLCL (B) Neat PEG (C) 40k/1k 90:10 PLCL/PEG blend and (D) 80k/3k 70:30 PLCL/PEG blend. Blending PLCL/PEG together results in C─O/C─OH bonds on the surface of the material, contributing to improved hydrophilicity.

To assess how blending PEG with PLCL modifies the surface chemistry of the materials, x‐ray photoelectron spectroscopy was performed. The neat PLCL C1s spectra is characterized by two principal peaks: the C‐C/C‐H peak at 284.4 eV and the O═C─O peak between 288–289 eV (Figure 4A). PEG should not possess this second peak and notably has a much higher C‐O/C‐OH peak compared to C‐C/C‐H (Figure 4B). The blended 40k/1k (90:10) reveals a stark change compared to either neat polymer. This blend is defined by three decomposed peaks, representing C‐C/C‐H at 284.4, C‐O/C‐OH at the expected ∼286 eV and the O═C─O peak between 288–289 eV (Figure 4C). Considering the staggering molecular weight and concentration differences between the two polymers, the intensity of the C‐O/C‐OH peak is a strong indicator of a significant portion of PEG being present on the surface of the material. This trend continues in the 80k/3k (70:30) polymer (Figure 4D). Additional polymer blends of PLCL/PEG show the same trend, indicating no apparent limitation on the distribution of PEG across the tested molecular weights and blend ratios (Figure S8). Because absolute photoelectron intensities can vary between polymer samples due to charging, roughness, and sampling geometry, we focus our interpretation on within‐spectrum peak assignments and relative peak‐area ratios rather than absolute counts.

**FIGURE 5:**
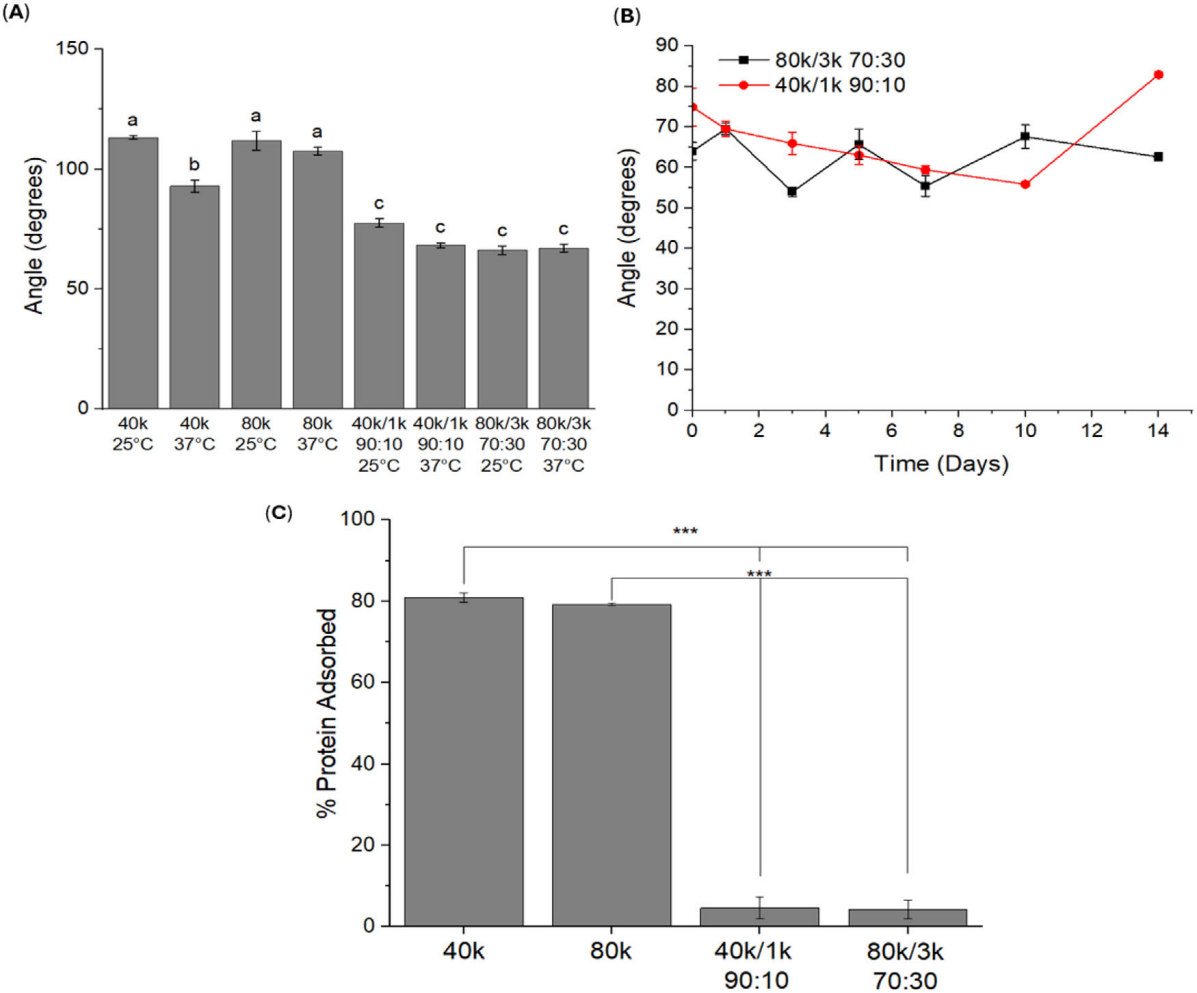
Surface characterization of sprayed polymer blends. (A) Day zero static water contact angles measured at room temperature and 37°C (body temperature) for neat PLCL (40k and 80k) and PLCL/PEG blends (40k/1k 90:10 and 80k/3k 70:30). (B) Changes in static contact angle during in vitro degradation of the polymer blends. (C) Protein adsorption profiles for neat PLCL compared with the PLCL/PEG blends. Data are reported as mean ± SE. Different letters denote statistically significant differences at a 0.01 significance level. Asterisks indicate statistical significance *p < 0.05; **p < 0.01; ***p < 0.001.

The XPS analysis revealed a much higher intensity of C─O/C─OH bonds in the PLCL/PEG blends on the material surface, which should result in improved hydrophilicity of the material. To confirm this, static water contact angle measurements were performed to determine the surface hydrophilicity of the sprayed blends. Immediately after spraying and incubation at 37°C, both the 80k/3k (70:30) and 40k/1k (90:10) blends demonstrated lower contact angles than neat PLCL (Figure 5A). Although the 80k/3k (70:30) blend contains nearly three times more PEG than the 40k/1k (90:10) formulation, both showed similar initial hydrophilicity. Interestingly, the 40k/1k (90:10) blend's contact angle decreased further after incubation, likely due to the 1 kDa PEG transitioning from fibers to a film at 37°C. By contrast, the 80k/3k (70:30) blend maintained a relatively constant contact angle during the same period. Extended degradation studies revealed that the 80k/3k (70:30) blend's contact angle remained around 60° for two weeks, whereas the 40k/1k (90:10) blend's contact angle gradually declined before showing a sudden increase between days 10 and 14 (Figure 5B). This can be attributed to a difference in diffusion rates of the 3 and 1 kDa PEG molecules from the bulk of the polymer matrix to the surface, wherein a higher concentration of PEG could be present in the polymer matrix in the 80k/3k (70:30) blend due to slower diffusion of 3 kDa PEG compared to 1 kDa PEG. Since the PEG molecules are blended into the polymer matrix without any covalent linkage, the diffusion of these molecules toward the surface in a swollen aqueous environment is to be expected although the rates can differ due to differences in PEG molecular weight and concentration in the blend. More importantly, we also see that the contact angle of both the blends remains around 60° throughout the first 7 days, indicating the presence of a consistently hydrophilic surface. This is important since the majority of adhesions form during the first week of wound healing and a hydrophilic barrier should theoretically prevent protein adsorption and consequently, postoperative adhesions. Protein adsorption assays further demonstrated that neat PLCL sprays adsorbed approximately 80% of available protein, whereas both PLCL/PEG blends displayed minimal adsorption (∼4%, Figure 5C). Sustaining this hydrophilicity throughout the early wound‐healing window, particularly the first two weeks, underscores the blends’ potential to reduce unwanted protein adsorption and, by extension, post‐operative adhesions.

### 3.2 In Vivo Efficacy in a Mouse Model of Abdominal Adhesions

Animal models for abdominal adhesions vary widely, and one commonly used approach involves forced serosal abrasion. However, the force applied by the operator can be inconsistent, potentially leading to variable abrasion severity and tissue area affected. For more reproducible results, it is critical to use a model with consistent injury parameters. One such method is the cecal ligation model, which standardizes the ischemic tissue injury and reliably induces robust adhesions. By ligating the distal end of the cecum, necrosis occurs, prompting a rapid and intense wound‐healing response that guarantees excessive adhesion formation within days. In this study, mice underwent cecal ligation before being randomized into groups that received no treatment (negative control), Seprafilm (clinical control), or SBS fibers composed of either 40 kDa PLCL/1 kDa PEG or 80 kDa PLCL/3 kDa PEG (treatment groups). Adhesion development and wound‐healing responses were assessed after eight days.

**FIGURE 6:**
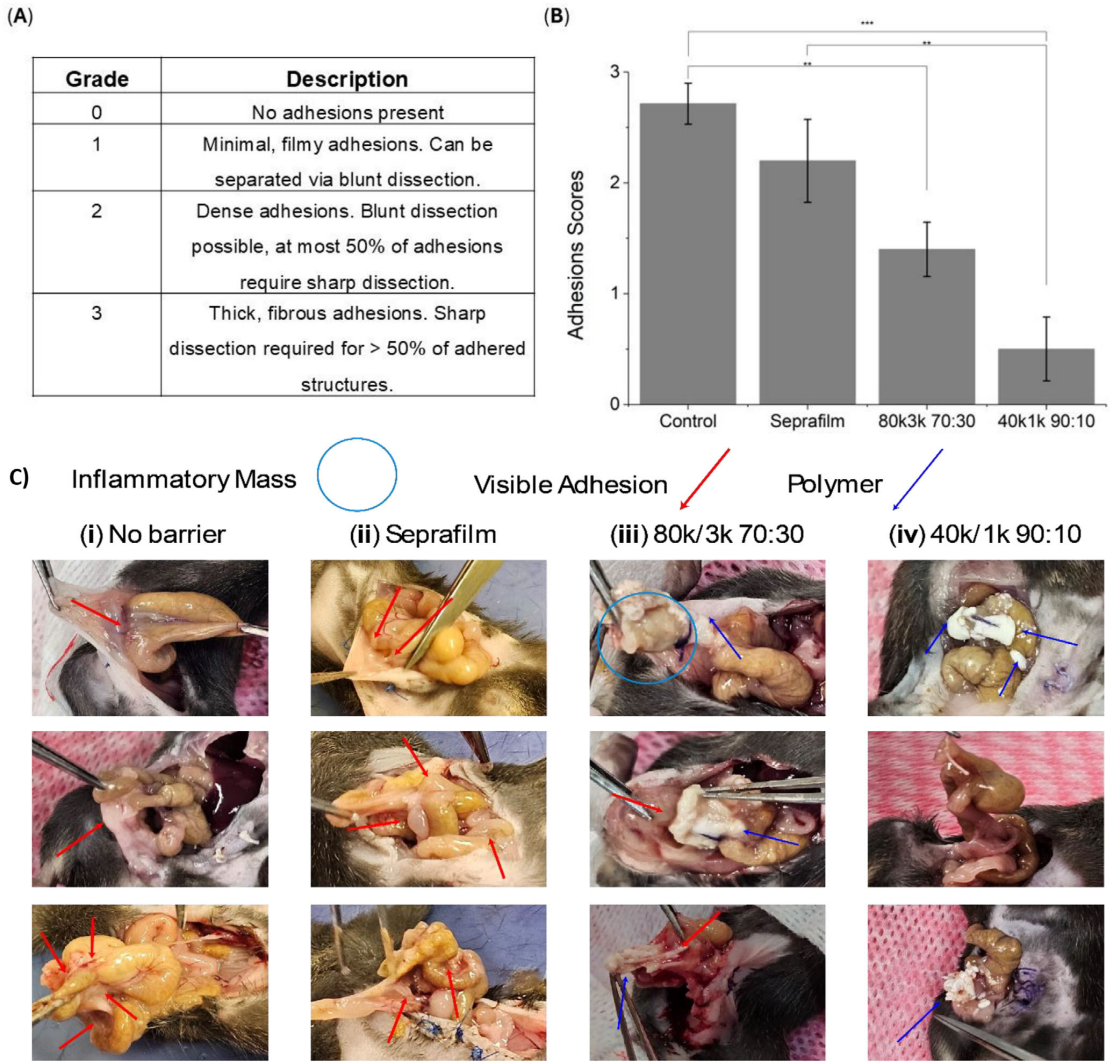
In vivo adhesions assessment. (A) Mazuji‐based scoring scale to evaluate adhesion severity. (B) Clinical adhesion scores and (C) gross pathology images at day eight post‐cecal ligation for (i) no barrier (negative control), (ii) Seprafilm (clinical control), (iii/iv) PLCL/PEG‐treated groups. The 40k/1k 90:10 blend demonstrated a significantly lower reduction in adhesion scores compared to both the negative and clinical controls. Data are shown as mean ± SE of the full n = 5 per group. Asterisks denote statistical significance: *p < 0.05; **p < 0.01; ***p < 0.001.

A surgeon, blinded to the treatment allocations, evaluated adhesion severity using a Mazuji‐based scoring system (Figure 6A) [46]. Both PLCL/PEG sprays showed markedly lower adhesion severity than untreated controls; notably, the 40k/1k formulation also performed significantly better than Seprafilm (Figure 6B,C). Further, the frequency of adhesions was substantially reduced in the 40k/1k group relative to both controls and the 80k/3k blend (Figure S9). The minimal adhesions observed in the 40k/1k‐treated group were noted to be localized and flimsy, and interloop adhesions were rarely found. In contrast, Seprafilm‐treated and negative control mice exhibited extensive adhesions involving the cecum, small bowel, omentum, and abdominal wall. Although the 80k/3k blend performed better than the negative control, its adhesion severity scores were inconsistent and not significantly different from Seprafilm. Consequently, this blend was excluded from further analyses. The superior performance of the 40k/1k formulation can be attributed to the lower melting point of 1 kDa PEG at body temperature, which produces a fiber‐to‐film transition and improves coverage. The resulting uniform, mechanically resilient, hydrophilic barrier effectively prevents protein adsorption and subsequent adhesions on the injured tissue.

**FIGURE 7:**
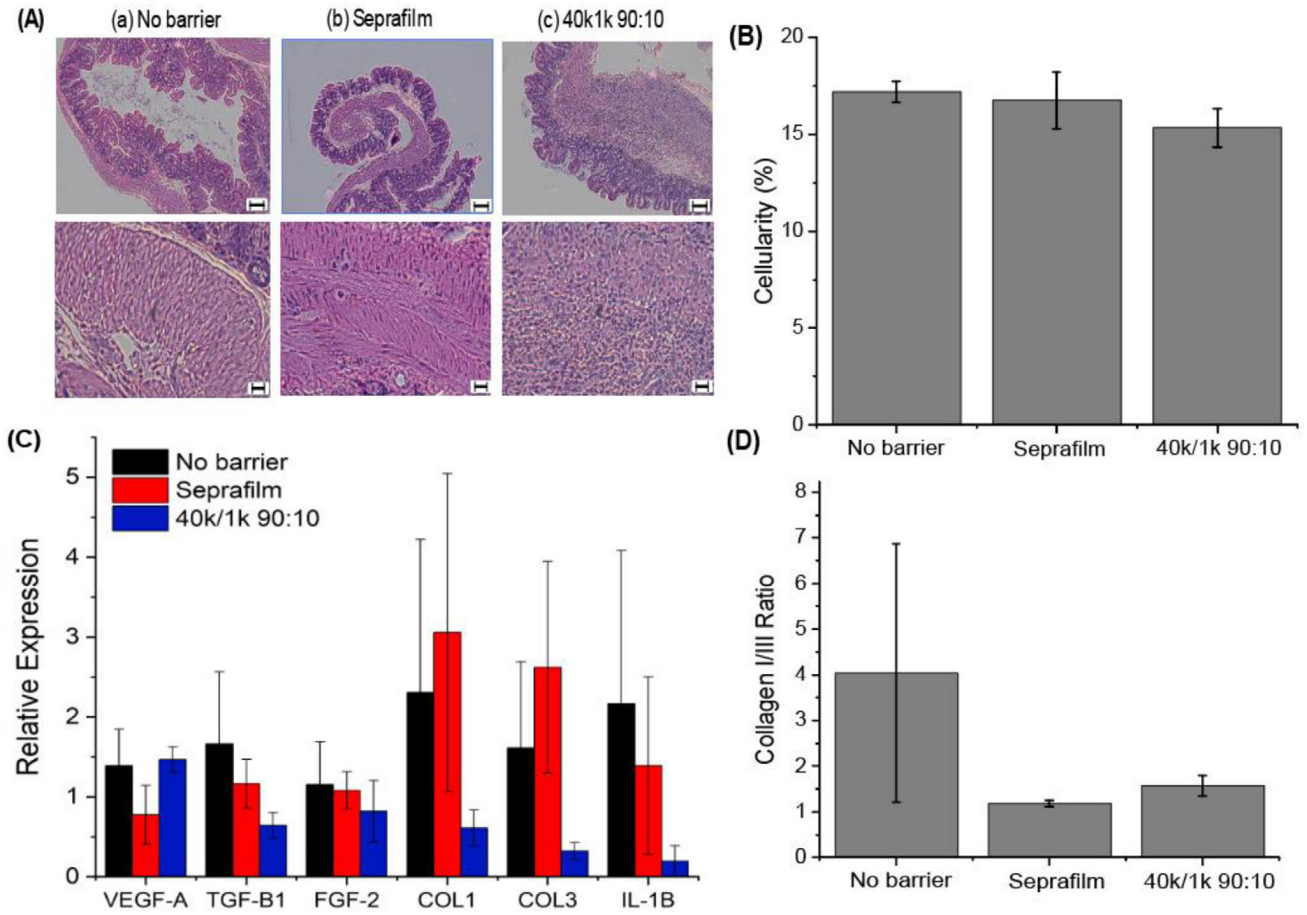
Histological and gene expression analysis. (A) Representative hematoxylin and eosin (H&E) cross‐sections of the mouse cecum and (B) corresponding cellularity measurements. (C) Relative mRNA expression levels of vascular endothelial growth factor (VEGF‐A), transforming growth factor‐β1 (TGF‐β1), fibroblast growth factor‐2 (FGF‐2), collagen I (COL1), collagen III (COL3), and interleukin‐1β (IL‐1β) were assessed via reverse transcriptase PCR, and (D) the ratio of collagen I to collagen III. Data are shown for (a) no barrier, (b) Seprafilm, and (c) 40k/1k (90:10) PLCL/PEG‐treated groups at 8 days post‐cecal ligation (n = 5). Top‐row scale bars represent 100 µm; bottom‐row scale bars represent 20 µm. Data are plotted as mean ± SE. Asterisks indicate statistical significance: *p < 0.05; **p < 0.01; ***p < 0.001.

To evaluate the wound‐healing response, cecal tissue was harvested on postoperative day eight for both gene expression and histology analyses. Histology serves a dual purpose: gauging inflammation (and correlating it to adhesion severity) and confirming that the sprayed polymers do not impede healing. Hematoxylin and eosin (H&E) staining revealed infiltration of eosinophils and neutrophils in the intestinal wall across all treatment groups (Figure 7A and Figure S10). However, cellularity analysis indicated no notable differences in overall inflammation (Figure 7B), suggesting that none of the sprayed blends triggered an elevated immune response. Gene expression levels of IL‐6, TNF‐α, and other inflammatory markers from ligated cecum samples were measured via RT‐PCR (Figure 7C and Figure S11). No significant changes emerged in any treatment group. Additionally, the collagen I‐to‐III ratio—a key indicator of scar tissue progression—was similar between the control groups and the 40k/1k spray group (Figure 7D). These findings collectively demonstrate that the presence of the polymer blends did not heighten inflammation or disrupt normal wound healing.

## 4 Discussion

With the biological complexity that governs adhesion formation, eliminating the practicality of therapeutic prevention methods, optimal clinical products that prevent adhesions should function as physical barriers. A physical adhesion barrier protects the wound surface from contacting surrounding tissues, thereby preventing adhesions. Consequently, materials employed for such barriers must be both biodegradable and biocompatible without disrupting the normal wound‐healing process. Equally important is their ease of application to minimize operative time and further reduce adhesion risk. In this regard, solution blow spinning with an airbrush provides a versatile method for depositing polymeric fibers onto the wound site of interest, conforming well to intricate tissue geometries. This technique also enables precise control over both the deposition rate and coverage area, ensuring adhesion of material to various tissue surfaces and allowing surgeons to cover larger surface areas for more severe wounds. Given the near guarantee for adhesions to form in abdominal surgery, a material that can be applied to any surface with any shape has become a clinical necessity. The strong tissue conformity and versatility to tailor the deposition of the sprayed material make SBS an exceptional application method for physical barriers to surgeons.

We introduce a sprayable polymer blend system designed to form fiber mats on tissue surfaces as a physical barrier against adhesions. The blends combine HMW PLCL with LMW PEG in ratios optimized to maintain both degradability and mechanical integrity. PEG is widely employed in biomaterial formulations to enhance biocompatibility and minimize protein adsorption, making it an ideal complement to PLCL [40, 41, 42, 43, 47, 48, 49]. We sought to leverage PEG's capacity to enhance anti‐fouling capabilities to minimize early‐stage adhesion formation. By introducing low molecular weight LMW PEG into our polymer blends, the materials effectively repel fibrinogenic and angiogenic factors, as corroborated by their decreased water contact angles, consistently maintained surface hydrophilicity throughout the critical wound healing period, and reduced protein adsorption (Figure 5A–C). This is attributed to the modified surface chemistry of the material upon blending LMW PEG (Figure 4C,D). Any in vivo barrier designed to keep tissues apart must adhere reliably to the treatment site while preserving sufficient mechanical strength throughout the wound‐healing window. Although Seprafilm exhibits good initial tissue adherence by converting into a hydrogel upon contact, this transformation leads to a pronounced loss in mechanical robustness [21, 50]. The PLCL/PEG sprays developed here demonstrate notably higher wet‐tissue adhesion—exceeding that of Seprafilm—while retaining most of their mechanical integrity through the course of degradation (Figure 2B,C). Mass loss studies indicate that the 40k/1k (90:10) blend preserves most of its mass, remaining as a film on the tissue surface throughout initial wound healing (Figure 2C). As a result, this hydrophilic physical barrier consistently separates injured tissue sites, preventing the build‐up of angiogenic and fibrinogenic proteins and thus impeding adhesion formation. We note that PEG is not covalently bound in these blends; therefore, some PEG redistribution and/or loss into the surrounding aqueous environment may occur. However, PEG can remain physically entrapped within the PLCL matrix and can dynamically enrich the surface during swelling and surface erosion, consistent with the sustained hydrophilicity observed over the early healing window. Methods to directly measure any PEG dissolution, such as gel permeation chromatography, cannot be applied to this system due to inherent insolubility of either polymer in aqueous or organic mobile phases. As such, exploring avenues to directly quantify PEG dissolution will be an important part of future work.

## 5 Conclusion

To conclude, both the 80k/3k (70:30) and 40k/1k (90:10) PLCL/PEG blends reduced adhesion formation compared to untreated controls in a cecal ligation mouse model. Notably, the 40k/1k formulation also outperformed Seprafilm in minimizing adhesion severity and frequency (Figure 6B,C, and Figure S8). Histological findings revealed no evident inflammation, consistent with the absence of elevated wound‐healing gene markers for all material groups. This indicates that neither Seprafilm nor the polymer sprays hamper the normal healing process or provoke an immunogenic response. The physical properties coupled with the exceptional in vivo performance of the PLCL/PEG blends presents a promising material‐based approach for preventing post‐operative adhesions.

## Author Contributions

**Robert J. Morris III**: Conceptualization, Methodology, Data Curation, Formal Analysis, Investigation, Visualization, Validation, Project Administration, Writing – Original Draft, Writing – Review and Editing **Tejaswi Nori** Conceptualization, Methodology, Investigation, Visualization, Project Administration, Writing – Original Draft, Writing – Review and Editing **Alex I. Halpern** Methodology, Investigation, Writing‐ Review and Editing **Hannah LaPadula** Investigation, Visualization, Writing – Review and Editing **Arthur V. Cresce** Methodology, Investigation, Writing‐ Review and Editing **Sarah L. Wright** Methodology, Investigation, Writing – Review and Editing **Anthony D. Sandler** Methodology, Funding Acquisition, Project Administration, Supervision, Writing – Review and Editing **Peter Kofinas** Conceptualization, Methodology, Funding Acquisition, Project Administration, Supervision, Writing – Review and Editing

## Funding

This work was supported by the National Institute of General Medical Sciences of the National Institutes of Health under Award Number R01GM141132.

## Conflicts of Interest

The authors declare no conflicts of interest.

## Supporting information




**Supporting File**: adhm71038‐sup‐0001‐SuppMat.docx.

## Data Availability

All data are available in the main text or the supplementary materials.

## References

[1] M. P. Diamond , “Incidence of Postsurgical Adhesions,” Peritoneal Surgery (Springer, 2000): 217–220.

[2] N. Tabibian , E. Swehli , A. Boyd , A. Umbreen , and J. H. Tabibian , “Abdominal Adhesions: A Practical Review of an Often Overlooked Entity,” Annals of Medicine & Surgery 15 (2017): 9–13, 10.1016/j.amsu.2017.01.021. 28203370 PMC5295619

[3] D. Menzies and H. Ellis , “Intestinal Obstruction From Adhesions–How Big is the Problem?,” Annals of the Royal College of Surgeons of England 72 (1990): 60. 2301905 PMC2499092

[4] C. I. W. Lauder , G. Garcea , A. Strickland , and G. J. Maddern , “Abdominal Adhesion Prevention: Still a Sticky Subject,” Digestive Surgery 27 (2010): 347–358, 10.1159/000314805. 20847564

[5] R. L. DeWilde and G. Trew , “Postoperative Abdominal Adhesions and their Prevention in Gynaecological Surgery. Expert Consensus Position,” Gynecological Surgery 4 (2007): 161–168, 10.1007/s10397-007-0338-x.

[6] M. A. Weibel and G. Majno , “Peritoneal Adhesions and their Relation to Abdominal Surgery,” The American Journal of Surgery 126 (1973): 345–353, 10.1016/S0002-9610(73)80123-0. 4580750

[7] D. Menzies , M. Parker , R. Hoare , and A. Knight , “Small Bowel Obstruction due to Postoperative Adhesions: Treatment Patterns and Associated Costs in 110 Hospital Admissions,” Annals of the Royal College of Surgeons of England 83 (2001): 40–46. 11212449 PMC2503561

[8] P. Capmas , F. Payen , A. Lemaire , and H. Fernandez , “Adhesions in Abdomino‐Pelvic Surgeries: A Real Economic Impact?,” PLoS ONE 17 (2022): 0276810, 10.1371/journal.pone.0276810. PMC9612443 36301908

[9] C. I. W. Lauder , G. Garcea , A. Strickland , and G. J. Maddern , “Abdominal Adhesion Prevention: Still a Sticky Subject,” Digestive Surgery 27 (2010): 347–358, 10.1159/000314805. 20847564

[10] K. J. Neis and F. Neis , “Chronic Pelvic Pain: Cause, Diagnosis and Therapy From a Gynaecologist's and an Endoscopist's Point of View,” Gynecological Endocrinology 25 (2009): 757–761, 10.3109/09513590903230366. 19908952

[11] H. Capella‐Monsonís , S. Kearns , J. Kelly , and D. I. Zeugolis , “Battling Adhesions: From Understanding to Prevention,” BMC Biomedical Engineering 1, no. 1 (2019): 1–12. 32903353 10.1186/s42490-019-0005-0 PMC7412649

[12] V. Gomel , B. Urman , and T. Gürgan , “Pathophysiology of Adhesion Formation and Strategies for Prevention,” Journal of Reproductive Medicine 41 (1996): 35–41. 8855074

[13] S. Buţureanu and T. Buţureanu , “Pathophysiology of Adhesions,” Chirurgia 109 (2014): 293–298. 24956331

[14] V. Sikirica , B. Bapat , S. D. Candrilli , K. L. Davis , M. Wilson , and A. Johns , “The Inpatient Burden of Abdominal and Gynecological Adhesiolysis in the US,” BMC Surgery 11 (2011): 1–9, 10.1186/1471-2482-11-13. 21658255 PMC3141363

[15] B. T. S. Fevang , J. Fevang , S. A. Lie , O. Søreide , K. Svanes , and A. Viste , “Long‐term Prognosis After Operation for Adhesive Small Bowel Obstruction,” Annals of Surgery 240 (2004): 193–201, 10.1097/01.sla.0000132988.50122.de. 15273540 PMC1356393

[16] R. P. G. Ten Broek , M. W. J. Stommel , C. Strik , C. J. H. M. Van Laarhoven , F. Keus , and H. Van Goor , “Benefits and Harms of Adhesion Barriers for Abdominal Surgery: A Systematic Review and Meta‐analysis,” The Lancet 383 (2014): 48–59, 10.1016/S0140-6736(13)61687-6. 24075279

[17] M. Nakashima , M. Takeuchi , and K. Kawakami , “Effectiveness of Barrier Agents for Preventing Postoperative Bowel Obstruction After Laparoscopic Surgery: A Retrospective Cohort Study,” Surgery Today 51 (2021): 1335–1342, 10.1007/s00595-021-02258-w. 33646411

[18] C. Farquhar , P. Vandekerckhove , A. Watson , A. Vail , and D. Wiseman , “Barrier Agents for Preventing Adhesions After Surgery for Subfertility,” Cochrane Database of Systematic Reviews (Online) 2 (2000): CD000475–CD000475. 10.1002/14651858.CD000475 10796548

[19] H. Saravelos and T. C. Li , “Post‐operative Adhesions After Laparoscopic Electrosurgical Treatment for Polycystic Ovarian Syndrome With the Application of Interceed to One Ovary: A Prospective Randomized Controlled Study,” Human Reproduction 11 (1996): 992–997, 10.1093/oxfordjournals.humrep.a019337. 8671376

[20] S. Hajibandeh , S. Hajibandeh , S. Saeed , J. Bird , L. K. Kannappa , and I. Ratnayake , “Effect of Hyaluronate‐based Bioresorbable Membrane (Seprafilm) on Outcomes of Abdominal Surgery: A Meta‐analysis and Trial Sequential Analysis of Randomised Controlled Trials,” Updates in Surgery 74 (2022): 865–881, 10.1007/s13304-021-01117-0. 34148173

[21] M. P. Diamond , E. L. Burns , B. Accomando , S. Mian , and L. Holmdahl , “Seprafilm Adhesion Barrier: (1) A Review of Preclinical, Animal, and Human Investigational Studies,” Gynecological Surgery 9 (2012): 237–245, 10.1007/s10397-012-0741-9. 22837732 PMC3401296

[22] B. Krämer , J. Andress , F. Neis , et al., “Adhesion Prevention After Endometriosis Surgery — Results of a Randomized, Controlled Clinical Trial With Second‐look Laparoscopy,” Langenbeck's Archives of Surgery 406 (2021): 2133–2143, 10.1007/s00423-021-02193-x. PMC8481146 34036409

[23] A. H. DeCherney and G. S. DiZerega , “Clinical Problem Of Intraperitoneal Postsurgical Adhesion Formation Following General Urgery And The Use Of Adhesion Prevention Barriers,” Surgical Clinics of North America 77 (1997): 671–688, 10.1016/S0039-6109(05)70574-0. 9194886

[24] H. Yao , Z. Cao , L. Peng , J. Liu , X. Zhang , and Z. Deng , “A Novel Controlled Release Tetrandrine‐loaded PDLLA film: Evaluation of Drug Release and Anti‐adhesion Effects In Vitro and In Vivo,” Drug Delivery and Translational Research 10 (2020): 13–22, 10.1007/s13346-019-00654-x. 31240625 PMC6978294

[25] X. Wang , X. Zhang , X. Yang , et al., “An Antibacterial and Antiadhesion In Situ Forming Hydrogel With Sol‐Spray System for Noncompressible Hemostasis,” ACS Applied Materials and Interfaces 15 (2023): 662–676. 36562696 10.1021/acsami.2c19662

[26] P. Lang , T. Liu , S. Huang , et al., “Degradable Temperature‐Sensitive Hydrogel Loaded With Heparin Effectively Prevents Post‐Operative Tissue Adhesions,” ACS Biomaterials Science & Engineering 9 (2023): 3618–3631, 10.1021/acsbiomaterials.3c00017. 37179492

[27] J. Yu , K. Wang , C. Fan , et al., “An Ultrasoft Self‐Fused Supramolecular Polymer Hydrogel for Completely Preventing Postoperative Tissue Adhesion,” Advanced Materials 33 (2021): 2008395. 10.1002/adma.202008395 33734513

[28] Y. Wang , L. Cheng , S. Wen , et al., “Ice‐Inspired Superlubricated Electrospun Nanofibrous Membrane for Preventing Tissue Adhesion,” Nano Letters 20 (2020): 6420–6428, 10.1021/acs.nanolett.0c01990. 32813534

[29] J. Zou , M. Lu , S. Chen , et al., “Beeswax‐inspired Superhydrophobic Electrospun Membranes for peritendinous Anti‐adhesion,” Materials Science and Engineering: C 116 (2020): 111166. 32806293 10.1016/j.msec.2020.111166

[30] J. L. Daristotle , L. W. Lau , M. Erdi , et al., “Sprayable and Biodegradable, Intrinsically Adhesive Wound Dressing With Antimicrobial Properties,” Bioengineering & translational medicine 5 (2020): 10149. 10.1002/btm2.10149 PMC6971445 31989038

[31] A. M. Behrens , N. G. Lee , B. J. Casey , et al., “Biodegradable‐Polymer‐Blend‐Based Surgical Sealant With Body‐Temperature‐Mediated Adhesion,” Advanced Materials 27 (2015): 8056–8061, 10.1002/adma.201503691. 26554545 PMC4961426

[32] A. M. Behrens , B. J. Casey , M. J. Sikorski , et al., “In Situ Deposition of PLGA Nanofibers via Solution Blow Spinning,” ACS Macro Letters 3 (2014): 249–254, 10.1021/mz500049x. 35590515

[33] N. G. Kern , A. M. Behrens , P. Srinivasan , et al., “Solution Blow Spun Polymer: A Novel Preclinical Surgical Sealant for Bowel Anastomoses,” Journal of Pediatric Surgery 52 (2017): 1308–1312, 10.1016/j.jpedsurg.2016.11.044. 27956071 PMC5459684

[34] J. L. Daristotle , S. T. Zaki , L. W. Lau , et al., “Improving the Adhesion, Flexibility, and Hemostatic Efficacy of a Sprayable Polymer Blend Surgical Sealant by Incorporating Silica Particles,” Acta Biomaterialia 90 (2019): 205–216, 10.1016/j.actbio.2019.04.015. 30954624 PMC6549514

[35] M. Erdi , S. Rozyyev , M. Balabhadrapatruni , et al., “Sprayable Tissue Adhesive With Biodegradation Tuned for Prevention of Postoperative Abdominal Adhesions,” Bioengineering & Translational Medicine 8 (2023): 10335, 10.1002/btm2.10335. PMC9842025 36684071

[36] M. Erdi , M. S. Saruwatari , S. Rozyyev , et al., “Controlled Release of a Therapeutic Peptide in Sprayable Surgical Sealant for Prevention of Postoperative Abdominal Adhesions,” ACS Applied Materials & Interfaces (2023), 10.1021/ACSAMI.3C00283/ASSET/IMAGES/LARGE/AM3C00283_0006.JPEG. PMC10485170 36884271

[37] R. J. Morris , T. Nori , A. D. Sandler , and P. Kofinas , “Postoperative Adhesions: Current Research on Mechanisms, Therapeutics and Preventative Measures,” Biomedical Materials & Devices 2024 (2024): 1–41. 10.1007/s44174-024-00236-7 PMC12381945 40881991

[38] B. C. Ward and A. Panitch , “Abdominal Adhesions: Current and Novel Therapies,” Journal of Surgical Research 165 (2011): 91–111, 10.1016/j.jss.2009.09.015. 20036389

[39] M. Abbas , A. E. Nafeh , M. Elsebae , and Y. Farouk , “Dose Related Effect of Systemic Antibiotics in Prevention of Postoperative Intra‐abdominal Adhesion Formation in Experimental Animals,” Journal of the Egyptian Society of Parasitology 38 (2008): 813–822. 19209764

[40] V. B. Damodaran and S. N. Murthy , “Bio‐inspired Strategies for Designing Antifouling Biomaterials,” Biomaterials Research 20 (2016), 10.1186/S40824-016-0064-4/ASSET/4605DA2C-1F04-482B-8644-078B3315BD59/ASSETS/GRAPHIC/S40824-016-0064-4.FIGURE006.GIF. PMC4913429 27326371

[41] S. Liu , W. Guo , S. Liu , and W. W. Guo , “Anti‐Biofouling and Healable Materials: Preparation, Mechanisms, and Biomedical Applications,” Advanced Functional Materials 28 (2018): 1800596.

[42] Q. Chen , D. Zhang , J. Gu , et al., “The Impact of Antifouling Layers in Fabricating Bioactive Surfaces,” Acta Biomaterialia 126 (2021): 45–62, 10.1016/j.actbio.2021.03.022. 33727195

[43] X. Zhou , Y. Wang , J. Ji , and P. Zhang , “Materials Strategies to Overcome the Foreign Body Response,” Advanced Healthcare Materials 13 (2024): 2304478, 10.1002/adhm.202304478. 38666550

[44] H. Yuk , C. E. Varela , C. S. Nabzdyk , et al., “Dry Double‐sided Tape for Adhesion of Wet Tissues and Devices,” Nature 575 (2019): 169–174, 10.1038/s41586-019-1710-5. 31666696

[45] R. Michel , L. Poirier , Q. Van Poelvoorde , J. Legagneux , M. Manassero , and L. Corté , “Interfacial Fluid Transport is a Key to Hydrogel Bioadhesion,” Proceedings of the National Academy of Sciences 116 (2019): 738–743, 10.1073/pnas.1813208116. PMC6338857 30602456

[46] M. K. Mazuji , K. Kalambaheti , and B. Pawar , “Prevention of Adhesions with Polyvinylpyrrolidone: Preliminary Report,” Archives of Surgery 89 (1964): 1011–1015. 14208444 10.1001/archsurg.1964.01320060079015

[47] M. Fujita , G. M. Policastro , A. Burdick , et al., “Preventing Post‐surgical Cardiac Adhesions With a Catechol‐functionalized Oxime Hydrogel,” Nature Communications 12 (2021): 1–14, 10.1038/s41467-021-24104-w. PMC8213776 34145265

[48] A. B. Kutikov and J. Song , “Biodegradable PEG‐Based Amphiphilic Block Copolymers for Tissue Engineering Applications,” ACS Biomaterials Science & Engineering 1 (2015): 463–480, 10.1021/acsbiomaterials.5b00122. 27175443 PMC4860614

[49] F. M. Veronese and G. Pasut , “PEGylation, Successful Approach to Drug Delivery,” Drug Discovery Today 10 (2005): 1451–1458, 10.1016/S1359-6446(05)03575-0. 16243265

[50] M. P. Diamond , E. L. Burns , B. Accomando , S. Mian , and L. Holmdahl , “Seprafilm Adhesion Barrier: (2) A Review of the Clinical Literature on Intraabdominal Use,” Gynecological Surgery 9 (2012): 247–257, 10.1007/s10397-012-0742-8. 22837733 PMC3401301

