# Supplementary material for: Sprayable Polymer Blends With Short‐Chain Surface Segregation for Preventing Postoperative Abdominal Adhesions
Source: Adv Healthc Mater. 2026 Mar 15;15(19):e05289. doi: 10.1002/adhm.202505289 (PMC13206380; doi:10.1002/adhm.202505289)
Supplement: Supplementary file 1 — Supporting File: adhm71038‐sup‐0001‐SuppMat.docx. [file ADHM-15-0-s001.docx]

**Supplementary Information**

**Sprayable Polymer Blends with Short-Chain Surface Segregation for Preventing Postoperative Abdominal Adhesions**

Robert J. Morris III^1^, Tejaswi Nori^1^, Alex I. Halpern^2^, Hannah LaPadula^1^, Arthur V. Cresce^3^, Sarah L. Wright^2^, Anthony D. Sandler^2^*, Peter Kofinas^1^*

^1^Department of Chemical and Biomolecular Engineering, University of Maryland, College Park, Maryland, USA

^2^Sheikh Zayed Institute for Pediatric Surgical Innovation, Joseph E. Robert Jr. Center for Surgical Care, Children's National Medical Center, Washington, District of Columbia, USA

^3^U.S. Army DEVCOM Army Research Laboratory, Battery Science Branch, Energy Sciences Division, Adelphi, Maryland, USA

**Materials and Methods**

***Burst pressure testing***

Porcine small intestine was first rinsed and rehydrated in PBS at 37°C, then gently dried at 37 °C before being cut into smaller segments of 10 cm in length. A 1 cm lateral incision was made using a scalpel, and both ends of the segment were sealed. The incision site was sprayed with 1 mL of polymer solution, or, for Seprafilm^®^, a 2 × 2 cm section of the clinical barrier was placed on the tissue. Following a 15-minute incubation at 37°C, the samples were injected with methylene blue–dyed PBS using a syringe. The peak burst pressure and mode of failure (adhesive or cohesive) were recorded. Each sealant formulation was tested five times (n = 5).

***Differential scanning calorimetry***

Polymer samples (~10 mg) were hermetically sealed in aluminum pans (TA Instruments) using an encapsulation press. Thermal analysis was performed on a DSC Q250 (TA Instruments). Each neat PEG sample was equilibrated at 0 °C for five minutes, then heated and cooled between 0°C and 80°C at a rate of 10 °C/min for two consecutive cycles. The melting temperature, T_m_, was determined from the maximum heat flow of the melting peak. For the blends, the procedure was the same with a new range of -80°C to 100°C to confirm the glass transition temperature of neat PLCL.

**
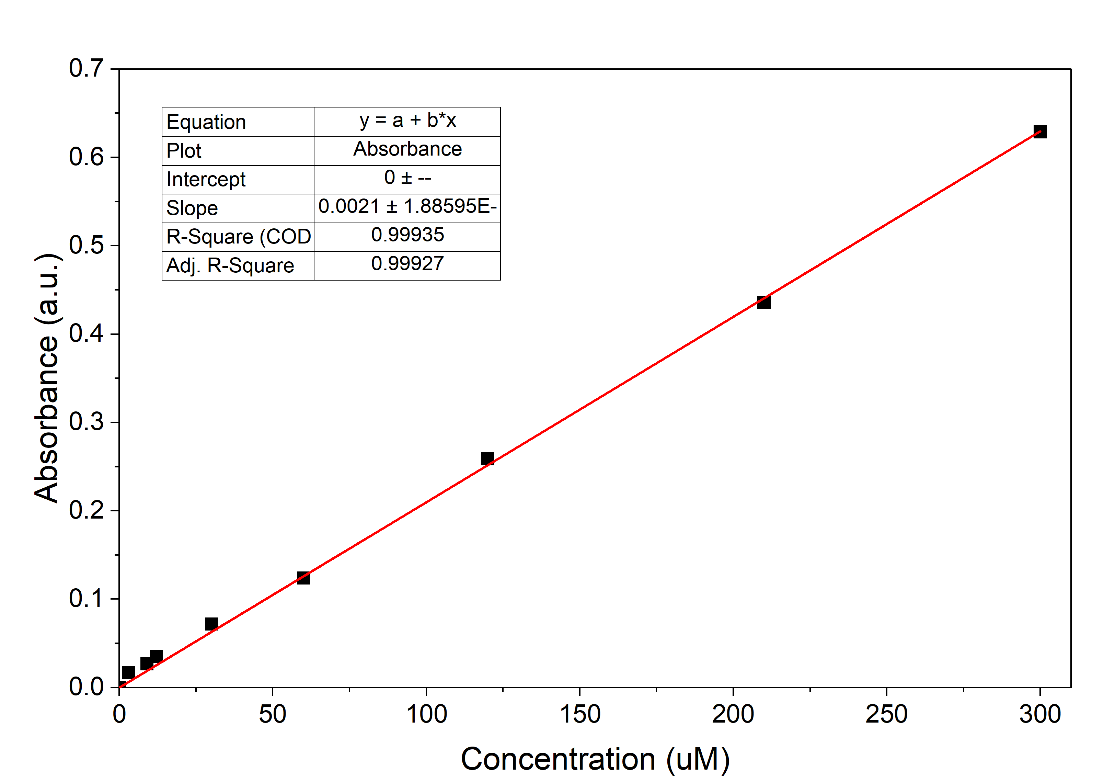
Figures:**

**Fig. S1. BSA Calibration Curve.** Protein absorption was calculated from this BSA standard curve. The y-intercept was fixed at zero and the absorbance of known concentrations of BSA were measured. The absorbance was then measured for the polymer sample supernatants and the background signal of protein solution in empty wells was subtracted. From the absorbance, the concentration of protein in solution was calculated using the calibration curve and this was converted to milligrams of BSA and subtracted from the initial mass of BSA added to each sample to determine how much protein was adsorbed onto the polymer.


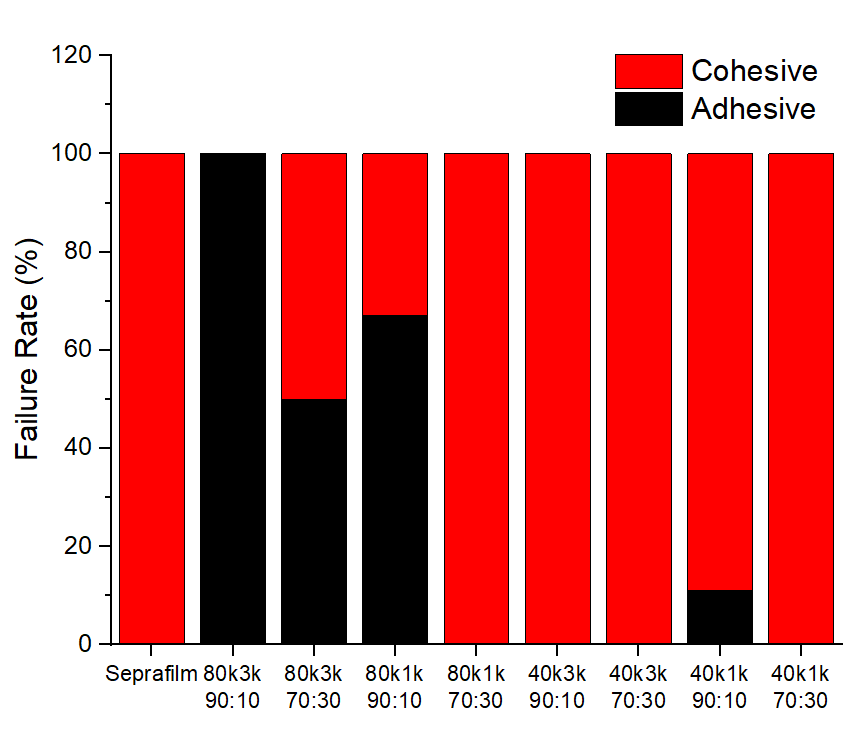


**Fig. S2. Failure Modes of Polymer Blends.** Adhesive versus cohesive failure events observed in pull-apart adhesion tests. The 40 kDa PLCL/PEG blends mostly failed cohesively, implying the polymer fibers ruptured before detaching from tissue. The 80 kDa blends exhibited mixed failure modes, while Seprafilm^®^ consistently failed cohesively.


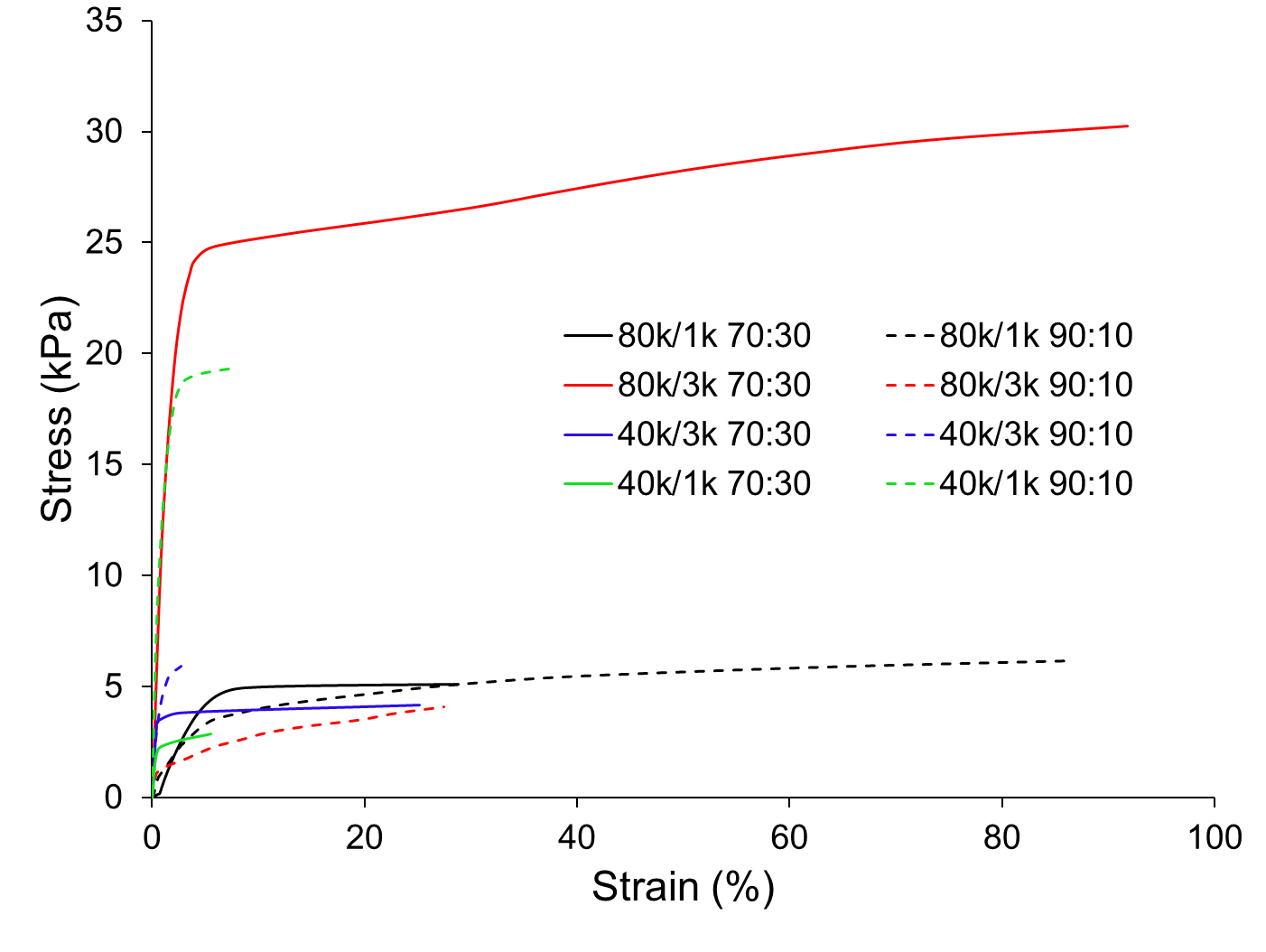


**Fig. S3. Representative Stress-Strain Curves.** Pull-apart adhesion tests result in these stress-strain curves, with the yield point representing reported values.

**
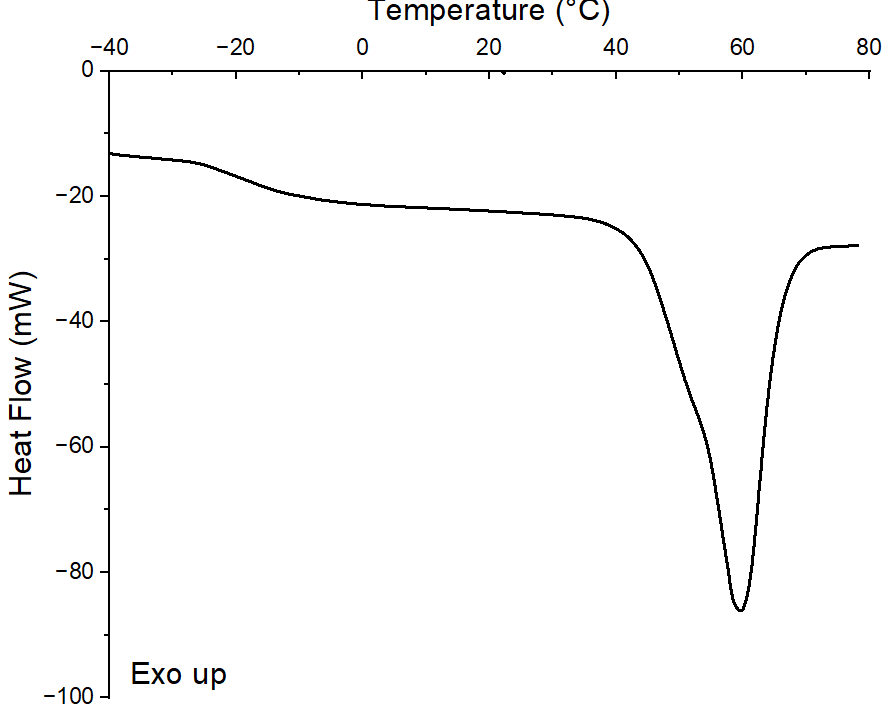
**
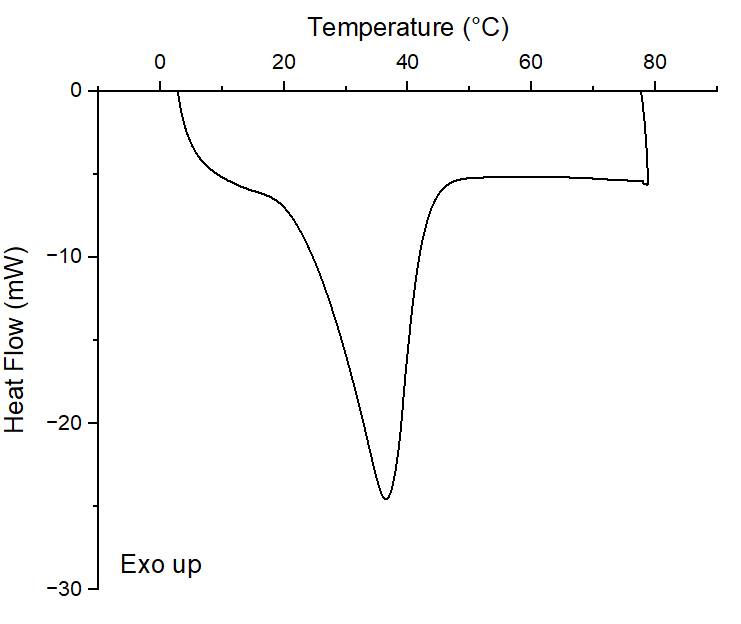
**
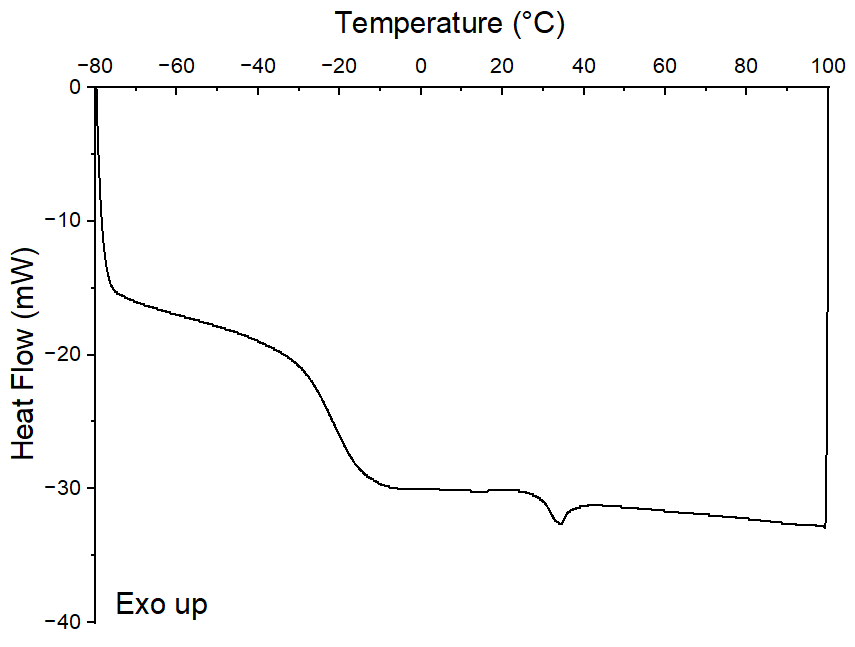
**
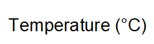

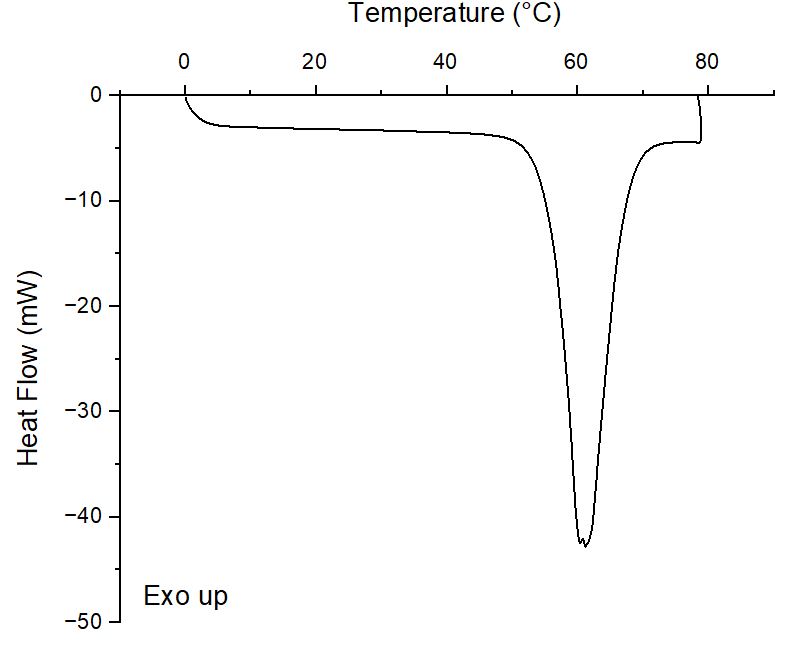


**(B)**

**(C)**

**(D)**

**(A)**

**Fig. S4. DSC Exotherms of neat PEG and PLCL/PEG blends.** (**A**) Differential scanning calorimetry exotherms of 1kDa PEG exhibiting melting around 37 °C. (**B**) Exotherm of 3kDa PEG with a melting peak at approximately 61 °C. (**C**) Exotherm of the PLCL/PEG blend 40k/1k 90:10, showing the glass transition of 40kDa PLCL and the melting peak of 1kDa PEG at approximately 34 °C. (**D**) Exotherm of the 80/3k 70:30 blend with a melting peak at approximately 60 °C.


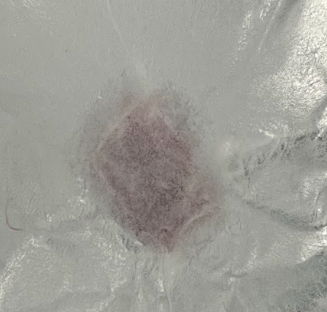

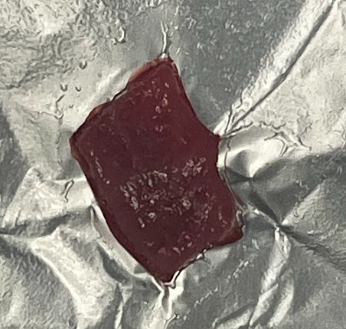


40k/1k 90:10 25°C

40k/1k 90:10 37°C


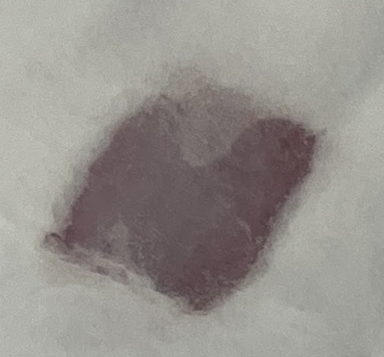


80k/3k 70:30 25°C


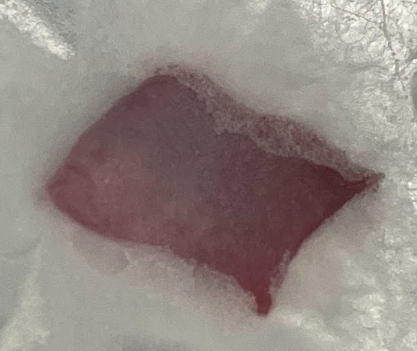


80k/3k 70:30 37°C

**Fig. S5. Visual Characterization of Selected Polymer Blends.** Photographs illustrating the 40k/1k (90:10) PLCL/PEG blend transitioning upon incubation at 37 °C. Blends containing 3 kDa PEG did not exhibit the same fiber-to-film transition, highlighting the impact of PEG molecular weight on polymer behavior.


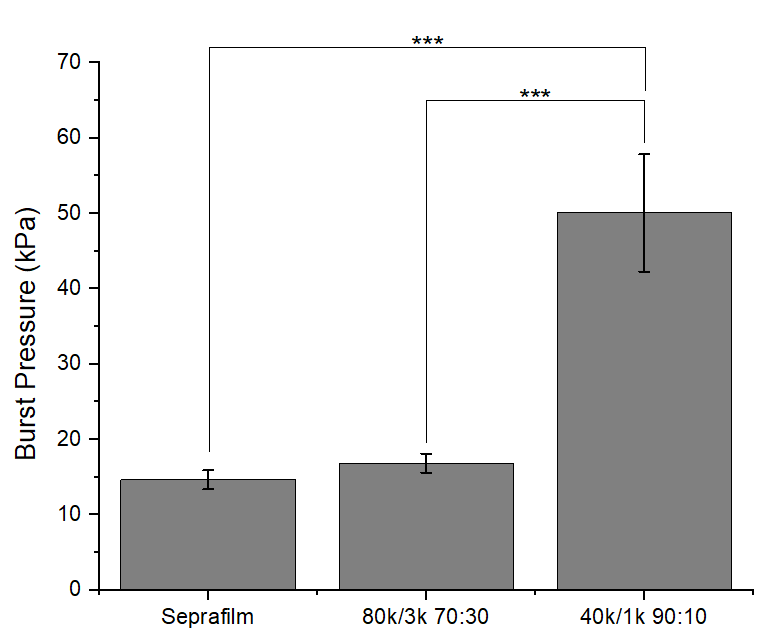


**Fig. S6. Burst pressure measurement.** The melting of 1 kDa PEG in the 40k/1k blend significantly enhanced surface conformity and sealing efficacy, as demonstrated by higher burst pressures. Seprafilm® is included for clinical reference. Data shown are mean ± SE, with asterisks indicating statistical significance (*p < 0.05; **p < 0.01; ***p < 0.001).


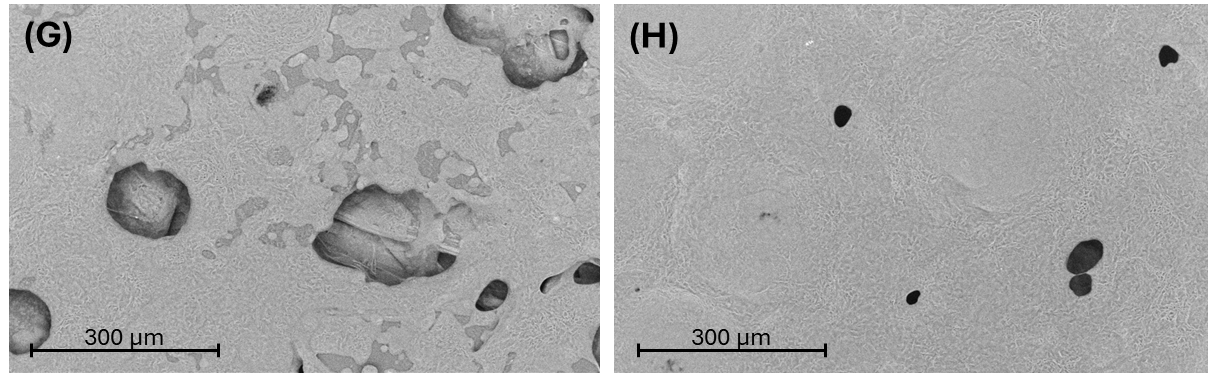

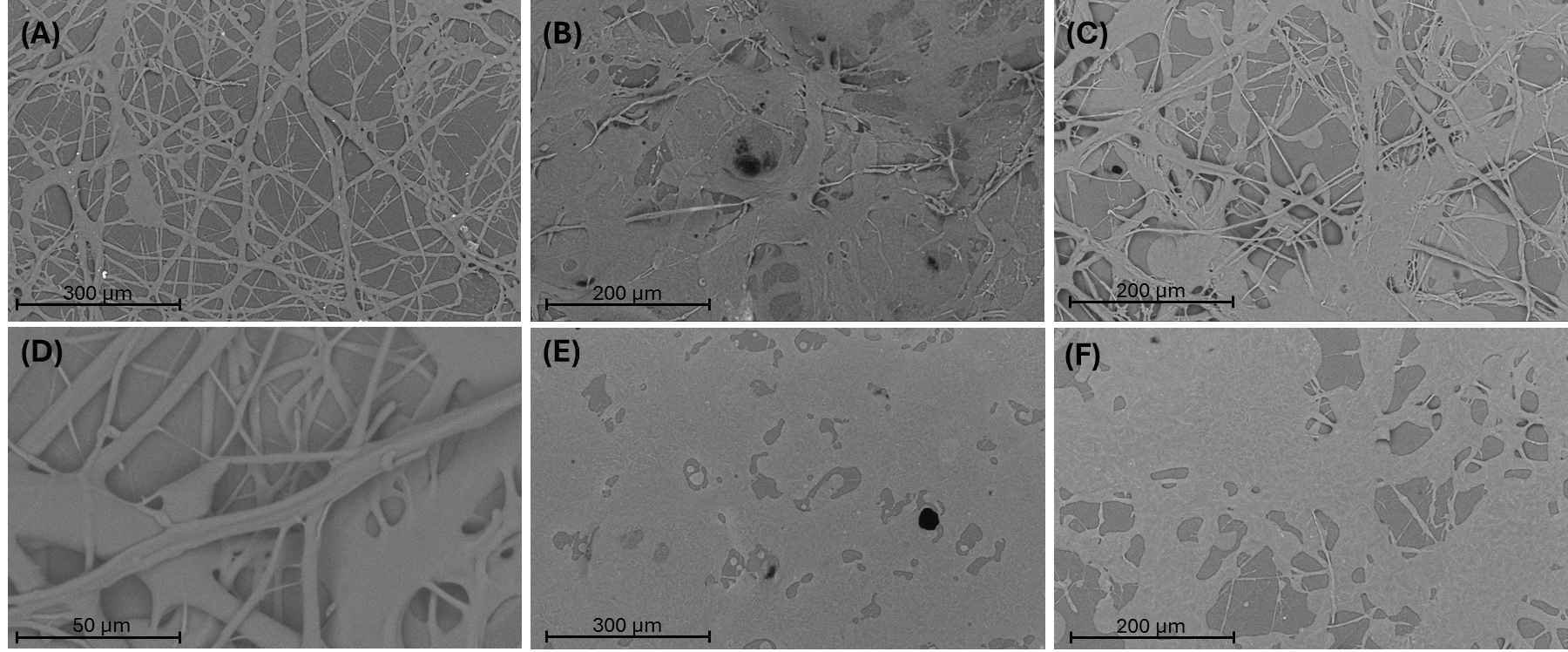


**Fig. S7. Additional SEM images of the polymer blends.** Microscopy images of (**A**) neat 40kDa PLCL, (**B**) 80k/3k (90:10), (**C**) 80k/1k (90:10), (**D**) neat 80kDa PLCL, (**E**) 40k/1k (70:30), (**F**) 80k/1k (70:30), (**G**) 40k/3k (90:10), and (**H**) 40k/3k (70:30). Inclusion of the 1kDa PEG results in a uniform film but, with the lower quantity of dense PLCL in 70:30 blends, it exhibits more apparent pores.


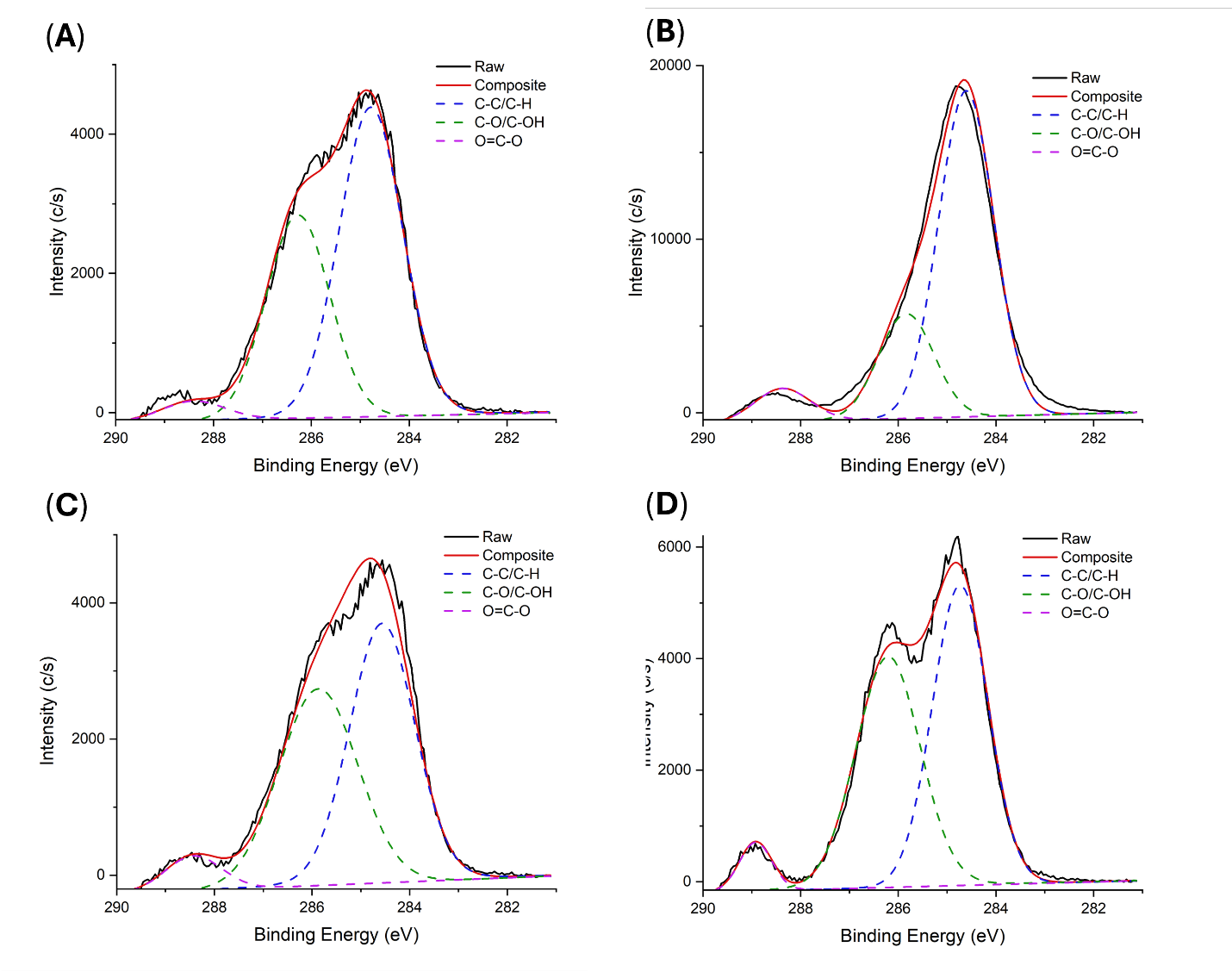


**Fig. S8. Additional XPS analysis of polymer blends.** XPS analysis of additional polymer blends which are (**A**) 40k/1k 70:30, (**B**) 80k/3k 90:10, (**C**) 40k/3k 90:10 and (**D**) 80k/1k 70:30 reveal that blending PEG, regardless of the two molecular weights and the concentration between 10-30% of the polymer in solution, results in increased intensity of the C-O/C-OH peak which is not present in neat PLCL.


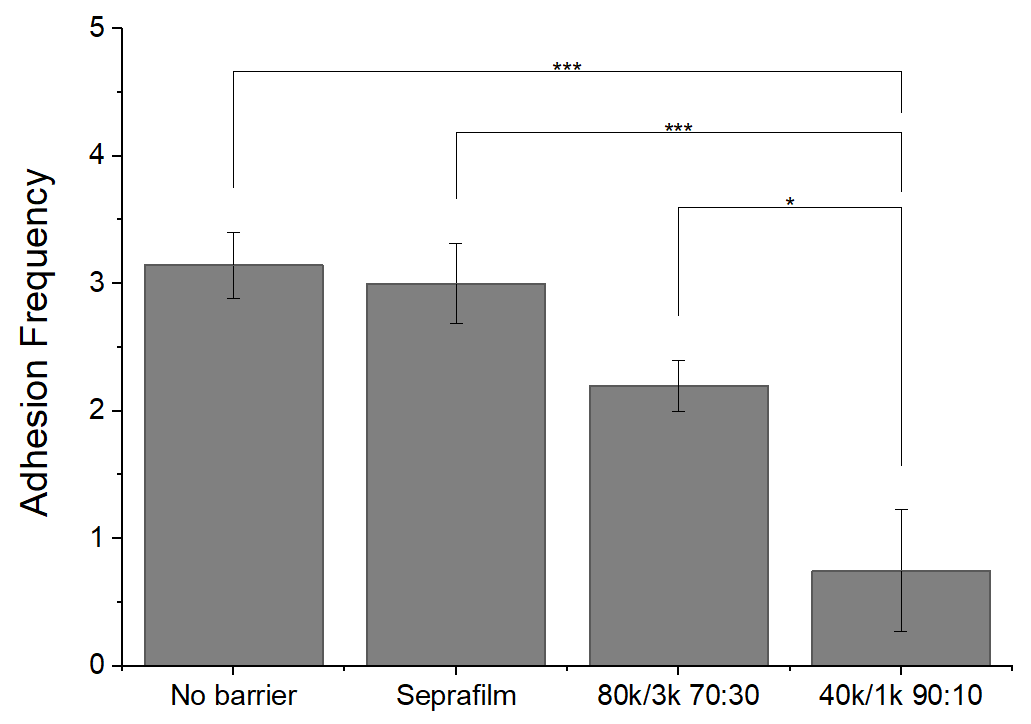


**Fig. S9. Adhesion frequency.** Total adhesions observed in the no-barrier (negative control), Seprafilm®, and PLCL/PEG-treated groups on day eight post-cecal ligation. Data are shown as mean ± SE; asterisks denote statistical significance (*p < 0.05; **p < 0.01; ***p < 0.001).

**Fig. S10. Additional Histologic Sections.** Representative H&E–stained images for mice treated with (a) no barrier, (b) Seprafilm®, and (c) 40k/1k 90:10 PLCL/PEG at t = 8 days post-cecal ligation. Scale bars represent 100 μm in top panels and 20 μm in bottom panels.


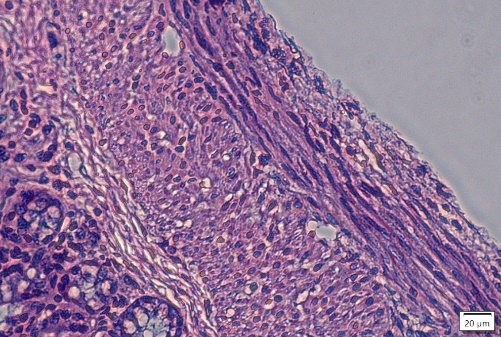

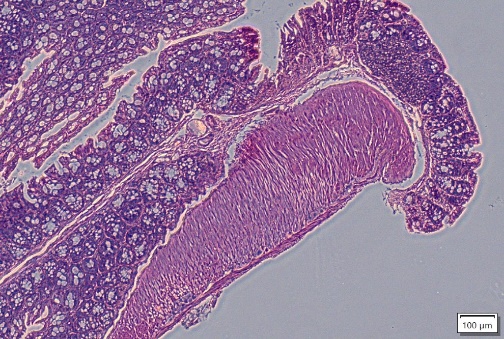

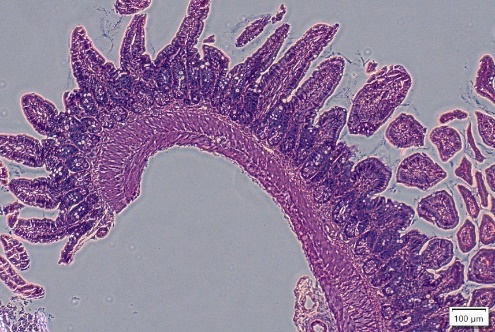

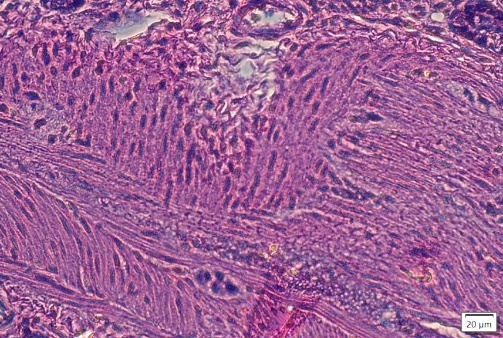

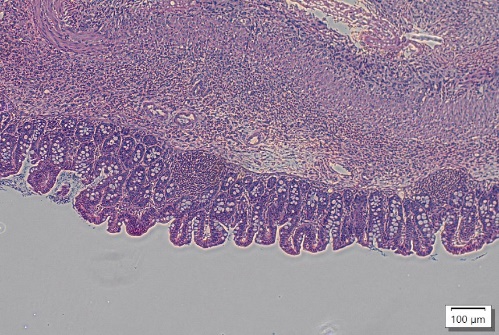

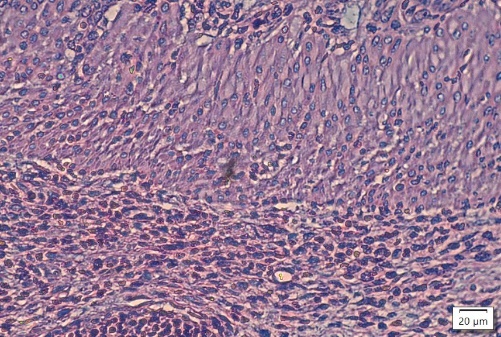


(a) No barrier

(b) Seprafilm

(c) 40k/1k 90:10


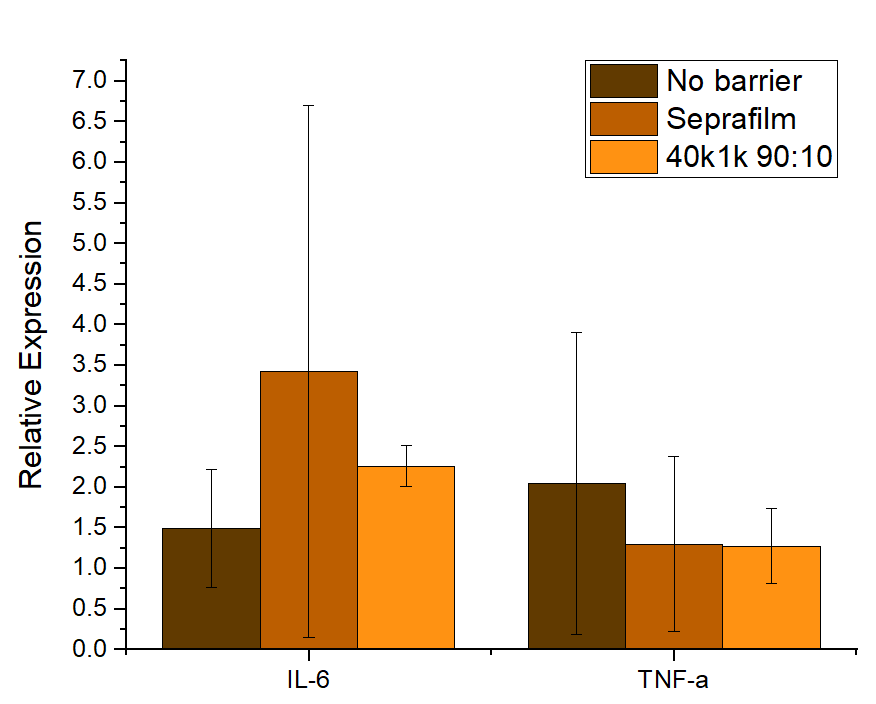


**Fig. S11. Additional mRNA expression levels.** RT-PCR analysis of IL-6 and TNF-α expression in cecal tissues from no-surgery controls, Seprafilm® controls, and 40k/1k 90:10 PLCL/PEG–treated mice at day 8 post-cecal ligation. Data are shown as mean ± SE. There is no statistical significance between the groups.
